# Adaptive plasticity and fitness costs of endangered, nonendangered, and invasive plants in response to variation in nitrogen and phosphorus availabilities

**DOI:** 10.1002/ece3.10075

**Published:** 2023-05-13

**Authors:** Vanessa Minden, Koen Verhoeven, Harry Olde Venterink

**Affiliations:** ^1^ Department of Biology Vrije Universiteit Brussel (VUB) Brussel Belgium; ^2^ Terrestrial Ecology Department Netherlands Institute of Ecology (NIOO‐KNAW) Wageningen The Netherlands

**Keywords:** adaptation, cost of plasticity, endangered species, nutrient limitation, phenotypic plasticity, plant functional traits, plant invasions

## Abstract

Global change drivers such as eutrophication and plant invasions will create novel environments for many plant species. Through adaptive trait plasticity plants may maintain their performance under these novel conditions and may outcompete those showing low‐adaptive trait plasticity. In a greenhouse study, we determined if plasticity in traits is adaptive or maladaptive in endangered, nonendangered, and invasive plant species in response to variation of nitrogen (N) and phosphorus (P) availability (N:P ratios 1.7, 15, and 135) and whether plastic trait responses are adaptive and/or costly for fitness (i.e., biomass). Species choice comprised 17 species from three functional groups (legumes, nonlegume forbs, and grasses), either classified as endangered, nonendangered, or invasive. After 2 months, plants were harvested and nine traits related to carbon assimilation and nutrient uptake were measured (leaf area, SLA, LDMC, SPAD, RMR, root length, SRL, root surface area, and PME activity). We found more traits responding plastically to variation in P than in N. Plasticity only created costs when P was varied. Plasticity in traits was mostly adaptively neutral toward fitness, with plasticity in three traits being similarly adaptive across all species groups: SPAD (as a measure of chlorophyll content, adaptive to N and P limitation), leaf area, and root surface area (adaptive to P limitation). We found little differences in trait plasticity between endangered, nonendangered, and invasive species. *Synthesis*. Along a gradient from N limitation, balanced N:P supply, and P limitation, we found that the type of fluctuating nutrient (i.e., if N or P is varied) is decisive for the adaptive value of a trait. Variation in P availability (from balanced supply to P limitation) created both a stronger reduction in fitness as well as created plasticity costs in more traits than variation in N availability (from balanced supply to N limitation). However, the patterns observed in our study may change if nutrient availability is altered, either by nutrient inputs or by a shift in nutrient availabilities, for example, by decreasing N input as foreseen by European Legislation, but without simultaneously decreasing P input.

## Graphical abstract

In the context of global change drivers, specifically eutrophication and exotic species invasion, we seek to answer if plasticity in plant traits is adaptive or maladaptive for fitness in endangered, non‐endangered and invasive congeneric plant species in response to variation of nitrogen (N) and phosphorus (P) availability. Our results indicate that although some plant traits responded plastically to nutrient availability, they did not contribute to plant fitness. We further show differences in trait plasticity and their effects on fitness between the type of nutrient that was varied (N and P), but they were similar between endangered, non‐endangered and invasive plant species.

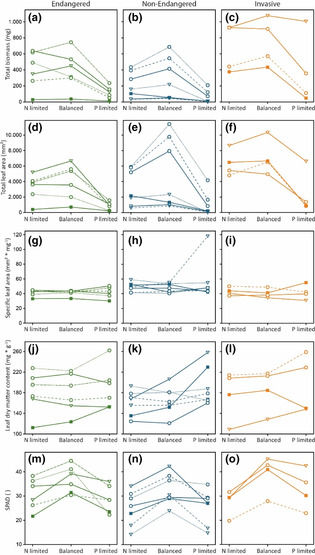

## 1 INTRODUCTION

Global change comprises anthropogenic environmental alterations that are, although occurring locally, so widespread that they scale to global significance (Matesanz et al., 2010). Various anthropogenic global change drivers have been defined (e.g., 10 by Sage, 2020, 5 by Sala et al., 2000, 4 by Millenium Ecosystem Assessment, 2005), among them exotic species invasions and ecosystem eutrophication. As both nitrogen (N) and phosphorus (P) are among the most limiting plant nutrients (Elser, 2011; Sterner & Elser, 2002), their massive increase in abundance in natural ecosystems through fertilizer use and industrial waste has led to significant losses of biodiversity on the level of primary producers (Bobbink et al., 2010; Simkin et al., 2016), upscaling on consumer levels, for example, by alteration of nutritional quality of plant tissue or availability of target plants (Nijssen et al., 2017; Stevens et al., 2018). A recent study estimated 18% of the natural terrestrial land area to be limited by N, 43% limited by P, and the remaining 39% either co‐limited by both N and P or weakly limited by either one of them (Du et al., 2020). Given strong nutrient inputs in many parts of the world, N:P imbalances are likely to increase, which may either result in higher abundance of P‐limited ecosystems (Crowley et al., 2012; Peñuelas et al., 2013; Tian et al., 2016), or cause a shift from P to N limitation, discriminating species adapted to low P availability (Wassen et al., 2005, 2021).

Especially N addition is seen to promote the spread of invasive species, another driver of global change (Daehler, 2003), whereas endangered species persist better under P limitation (Fujita et al., 2014; Wassen et al., 2005). Species are considered invasive when they were introduced to a novel range and produced reproductive offspring in areas distant from their sites of introduction (Richardson et al., 2000). A meta‐analysis by van Kleunen et al. (2010) concluded that invasive species showed higher values related to performance, measured, for example, as high leaf nitrogen content, specific leaf area (SLA), photosynthetic rate, or shoot root ratio, than noninvasive species. In contrast, endangered species are often small in stature and as such are poor competitors for light (Fujita et al., 2014). They further show a lower investment in sexual reproduction than, for example, common species (Fujita et al., 2014 after, Murray et al., 2002). Low competitive ability is a major disadvantage in fertile habitats, but not necessarily in unfertile habitats, such as P‐limited systems. Enhanced N supply has reportedly promoted the abundance of nonnative plants in various ecosystems, for example, coastal estuaries (Tyler et al., 2007), grasslands (Olson & Blicker, 2003), deserts (Brooks, 2003) or tropical forests (Ostertag & Verville, 2002), and enhanced P supply stimulated alien plants in the Brazilian Cerrado (Lannes et al., 2016). The effects of nonnative species are numerous and include effects on nutrient cycling and hydrology in ecosystems, changes in species composition, and effects on ecosystem functioning (Hejda et al., 2009; Pyšek & Richardson, 2010). In Europe, approximately 5200 plant species have been classified as alien with estimated economic costs of €116.6 billion between 1960 and 2020, the majority being damage‐related (Arianoutsou et al., 2021; Haubrock et al., 2021).

Traits of invasive species are often described as phenotypically more plastic than those of their noninvasive or native congeners (Daehler, 2003), but see Leishman and Thomson (2005) and Palacio‐Lopez and Gianoli (2011) for examples of similar trait plasticity between invasive and noninvasive plants. Plasticity is the capacity of a single genotype to produce different phenotypes in different environments (Sultan, 2000), and it decreases from nutrient‐poor to nutrient‐rich habitats (Aikio & Markkola, 2002). When plasticity in certain traits positively affects fitness, it is considered adaptive (Sultan, 1995). This can be achieved by either maintaining fitness in a stressful environment or, in a new environment, reducing the loss in fitness or achieving greater fitness, respectively (Sultan, 1995). Adaptive plastic responses of traits have been reported from many unfavorable environments such as nutrient poor or dry habitats (Cenzano et al., 2014; Heschel et al., 2004; Hodge, 2004). They range from changes on plant organ levels, such as changes in leaf stomatal conductance or increases in root length (Douhovnikoff et al., 2016; Heschel et al., 2004) to adjustments on the whole plant level, such as growth patterns or biomass allocation (Matesanz et al., 2010). However, plasticity can be costly when “‘in a focal environment a plastic organism exhibits lower fitness while producing the same mean trait value as a nonplastic organism” (DeWitt et al., 1998; van Kleunen & Fischer, 2005). Further, when plastic responses reduce fitness, they are considered to be maladaptive (Palacio‐Lopez et al., 2015; Valladares et al., 2007). For example, root‐secreted phosphatase activity is related to the plant's ability to make soil P available for absorption and can be adaptive under P limitation (Machado & Furlani, 2004). However, as its production is costly in N, it may be maladaptive under N limitation, leading to weaker competitive performance as shown for *Agrostis capillaris* (Olde Venterink & Güsewell, 2010).

Global change effects will comprise the creation of new environmental scenarios, including some that plants may not have experienced before (Alexander et al., 2015; Matesanz et al., 2010). Plants may maintain their performance in those novel environments through plastic trait responses, but only if plasticity is adaptive, that is, positively affects fitness (Richards et al., 2006). Through adaptive plasticity, some species may colonize these “new” habitats without the lag time needed for local adaptation. This in turn may enhance the invasiveness of some species and their potential to displace others that show low adaptive trait plasticity (Sultan, 2004). Although studies have demonstrated plastic responses of plant traits toward fluctuating nutrient availability (Hodge, 2004; Hodge et al., 2000), a systematic evaluation of the adaptive value of plasticity and plasticity costs on fitness in the framework of nutrient availability is pending.

In this study, we examined through greenhouse experiments if plasticity in traits is adaptive or maladaptive in endangered, nonendangered, and invasive congeneric plant species in response to variation of N and P availability (i.e., N limitation, balanced nutrient supply, and P limitation). Plants respond differently to N or P limitation, as they take different functions in plants: N is a constituent of enzymes and other proteins, nucleic acids, and chlorophyll (Marschner, 2012). Under N limitation, total root length is increased as is root surface area and transpiration rate, the latter to promote uptake of N (Garnett et al., 2009; Olde Venterink & Güsewell, 2010). P is essential for energy transport and is integral to cell membranes, DNA and RNA. When P is limiting, plants show reduced shoot growth, greater root length/mass quotient (i.e., specific root length, SRL), higher investment into phosphatases to degrade organic P compounds, impaired reproduction, and premature senescence in leaves (Holdaway et al., 2011; Marschner, 2012; Olde Venterink & Güsewell, 2010; White et al., 2013). Under both N and P limitations, plant performance is hampered, also because of the strong functional links between N and P: whereas N is an integral part of the photosynthetic enzyme Rubisco, P is required for its synthesis (Sterner & Elser, 2002). In our study, we measured both plant traits related to fitness (i.e., biomass production) and those related to direct responses to nutrient availability, such as root mass ratio (RMR), specific root length (SRL), and root phosphatase activity.

We hypothesized that traits of invasive species are more plastic than those of their nonendangered or endangered counterparts and that endangered species are the least plastic in their traits. Similarly, we expected invasive species to show the highest and endangered species to show lowest trait adaptability. Last, we aimed on identifying traits that show high plastic responses toward fluctuations in either N or P availability. For example, a high root length is advantageous under N limitation (Linkohr et al., 2002), as is a high phosphatase activity under P limitation (Minden & Olde Venterink, 2019; Olde Venterink & Güsewell, 2010). In reverse, high plasticity in these traits within (e.g., within each N‐ or P‐limited treatment) and across environments (e.g., across balanced nutrient supply and N‐ or P‐limited treatments) may positively affect fitness, although this has not been tested before.

## 2 MATERIALS AND METHODS

### 2.1 Study species

The experiment was conducted with 17 species of three families (Fabaceae, Onagraceae, and Poaceae) comprising three functional groups (nonlegume forbs, grasses, and legumes). Plants from different functional groups respond differently to variations in N and P. For example, legumes show higher phosphatase activity under low P availability than nonlegume forbs, whereas phosphatase activity under high N supply is similar between legumes and nonlegume forbs (Olde Venterink, 2011b). Effects of nutrient availability on biomass production have been shown by Sui et al. (2011), who found higher biomass production in the legume *Hedysaria laeve* than in the nonlegume forb *Artemisia ordosica* under low levels of N, P, and K (potassium). Species choice included their “status” of endangered, nonendangered, and invasive species (Table 1, for more detailed information about their range of origin and introduction, see Table S1). Prior to the main experiment, all species were raised in standard potting soil in the greenhouses of the Vrije Universiteit Brussel (VUB), Belgium, until they produced seeds. This was done to account for potential differences in the parental environments of the seed‐growing companies (Latzel, 2015). For *Lupinus angustifolius* and *Trifolium subterraneum*, however, seed output from individuals in potting soil was too small, so that seeds from the seed suppliers had to be used.

**TABLE 1:** Species list of this study and their classification as endangered (IUCN Red List, 2021) or invasive (Richardson et al., 2000).

	Onagraceae (nonlegume forbs)	Poaceae (grasses)	Fabaceae (legumes)
Endangered	^1^ *Epilobium fleischeri* Hochst.	^4^ *Bromus secalinus* L. ^3^ *Bromus squarrosus* L. ^3^ *Lolium remotum* Schrank ^2^ *Lolium temulentum* L.	^6^ *Trifolium subterraneum* L.
Nonendangered	^2^ *Epiobium anagallidifolium* Lam.	^4^ *Bromus hordeaceus* L. ^3^ *Bromus japonicus* Houtt. ^5^ *Hordeum murinum* L.	^2^ *Medicago lupulina* L. ^4^ *Trifolium arvense* L. ^4^ *Trifolium dubium* Sibth.
Invasive	^3^ *Epilobium ciliatum* Raf.	^2^ *Avena sterilis* L. ^2^ *Hordeum jubatum* L.	^7^ *Lupinus angustifolius* L.

*Note*: Seed suppliers: ^1^Jelitto Samen, Germany; ^2^B&T Seeds, France; ^3^Botanical Garden Konstanz, Germany; ^4^Rieger‐Hofmann, Germany; ^5^WeberSeeds, The Netherlands; ^6^Templiner Kräutergarten, Germany; ^7^Kiepenkerl, Germany.

### 2.2 Experimental design

Seeds of each species were sterilized prior to sowing to prevent seed‐borne plant diseases. Seeds were soaked in ethanol (70%) for 1 min and then 5 min in sodium hypochlorite (5%) with a drip of Tween® 20, after which they were rinsed five times with Aqua_dest_. Seeds were germinated on filter paper in a climate chamber at 25°C at approximately 46% humidity and a 16/8 h day/night rhythm. When radicula and cotyledons were visible, seedlings were transferred to a pot filled with quartz sand (without N or P) until they were big enough to be transferred to their experimental pots (between 7 and 41 days after sowing, depending on species). Each experimental pot (10 cm high, 8 cm wide) was filled with 500 mL fine quartz sand and contained one plant individual. Only pots of *Lu. angustifolius* were bigger (16 cm high, 15 cm wide, 3000 mL), because of their long primary roots. Sand was ordered from Sibelco Benelux (type M31, containing 99.5% SiO_2_, 0.04% FeO_3_, 0.2% Al_2_O_3_, 0.03% TiO_2_, 0.03% K_2_O, 0.01% CaO, and with a pH of 7) and analyzed amounts of N and P were below detection limits (analyzed with continuous flow analyzer [CFA], Egnér et al., 1960, Murphy & Riley, 1962).

The experiment started from mid‐April 2021 (earliest for *Avena sterilis*) to mid‐December 2021 (latest for *Trifolium subterraneum*). There was no systematic difference in the growing period among the three functional groups, meaning species of each group were grown at all times during the whole course of the experiment. In each treatment, 10 replicates per species were used, which ended up with a total of 510 plant individuals (17 species × 3 treatments × 10 replicates). Three treatments were applied: N limitation, balanced nutrient supply, and P limitation. Each plant individual of the N‐limited treatment received a total amount of 13.5 mg N and 8.1 mg P, which corresponds to an N:P ratio of 1.7. Individuals of the balanced nutrient supply received a total of 40.5 mg N and 2.7 mg P (N:P ratio of 15) and those of the P‐limited treatment a total of 121.5 mg N and 0.9 mg P (N:P ratio of 135). The N:P ratios of this study follow the designs of Güsewell (2005a) and Olde Venterink and Güsewell (2010), who included an N:P ratio of 15 as the balanced N and P treatment, whereas the N:P ratio of 1.7 and 135 showed the strongest effects at N‐ or P‐limited conditions, respectively.

N was supplied as NaNO_3_ and P as NaH_2_PO_4_.2H_2_O (details on concentrations of all macro‐ and micronutrients applied are provided in Table S2). pH of nutrient solutions was adjusted to 5.5 and each plant individual received 5 mL of nutrient solution per week. All other macro‐ and micronutrients were supplied in the same amounts (total per pot: 444 mg K [as KCl], 110 mg Ca [as CaCl_2_·2H_2_O], 41 mg S [as MgSO_4_·7H_2_O], 63 mg Mg [MgSO_4_·7H_2_O], 10 mg Fe [FeSO_4_·7H_2_O], 0.8 mg B [H_3_BO_3_], 0.06 mg Cu [CuSO_4_·5H_2_O], 0.5 mg Mn [as MnCl_2_·4H_2_O], 0.2 mg Zn [ZnSO_4_·7H_2_O], 0.1 mg Mo [as Na_2_MoO_4_·2H_2_O]). The designs of the nutrient solutions followed Güsewell (2005b) and (2005a, see appendix 1 of the article for nutrient concentrations). We adjusted the experimental design of Güsewell (2005a) who applied a high‐ and a low‐nutrient supply level and a total duration of 16 weeks on perennial sedges. We recalculated the nutrient concentrations to apply to an intermediate supply level (half of the sum of the total amount provided by high and low supply levels) and shortened the duration of the experiment for each species to 8 weeks, as our experimental plants were annual, not perennial.

To account for accumulating salt concentration in the pots, each pot was rinsed after 4 weeks (half of the experimental time). Saucers were removed and pots were filled up three times with distilled water until all the water was drained out, then clean saucers were placed underneath each pot. Watering of the plants during the course of the experiment was done when the upper layer of sand and the saucers were dry (appr. 2 times per week). To avoid the leaching of nutrients from the sand, de‐ionized water was only filled in saucers. Pots were randomized within species batches of 30 pots and regularly rearranged to avoid edge and/or shading effects.

### 2.3 Harvest and measurement of plant traits

On the day of harvest (i.e., after 8 weeks), SPAD values as a measure of chlorophyll content were determined from two leaves of each plant individual. For this, three measurements per leaf were taken with a Chlorophyll Meter 502‐SPAD Plus (Konica Minolta). The same leaves were weighed in their fresh state, their area was determined with a flatbed scanner and the computer software Image J (Rasband, 1997–2018) and then they were dried and weighed again (70°C, 72 h). From this, specific leaf area (SLA, without petiole, area of a fresh leaf divided by its dry weight, mm^2^ mg^−1^) and leaf dry matter content (LDMC, without petiole, dry weight of a leaf divided by its fresh weight, mg g^−1^) were determined. Total leaf area (mm^2^) refers to the area of the total leaf mass of living leaves at the time of harvest.

During the course of the experiment and on the day of harvest, dead biomass, consisting mainly of dead leaves, was collected. The remaining plant parts were harvested. The sand was washed off the roots and individuals were separated into roots, stems, leaves, and flowers, and/or seeds where applicable. Leaves consisted of the leaf blades, whereas petioles were considered part of the stems. We decided on this to be consistent across functional groups, as in grasses leaf petioles contribute significantly to their upright stability and function as part of the stem. All biomass samples were dried at 70°C for 72 h and weighed. Root mass ratio (RMR, mg mg^−1^) was calculated as the ratio of root mass to total biomass (including dead biomass, Pérez‐Harguindeguy et al., 2013).

From all harvested plant individuals, a part of the fresh root mass was used to determine total root length (m), specific root length (SRL, ratio of root length to its dry mass, m mg^−1^), and root phosphomonoesterase activity (PME activity, μmol pNPP cleaved per g fresh root per hour, μmol pNPP g^−1^ h^−1^). For PME activity, 100 mg fresh root mass, clipped off from the top of the root to represent its whole length, was processed with para‐nitrophenyl phosphate and tris(hydroxymethyl‐)aminomethane/maleate (Hogan et al., 2010; Olde Venterink & Güsewell, 2010), with consecutive measurement of the absorbance at 410 nm (Genesys™ 10 Series Spectrophotometer; Thermo Spectronic); for further method description, see Olde Venterink (2011a). For SRL, another subsample of roots was clipped off in the same way as for PME activity and scanned (LA2400 scanner for WinRhizo®, Regent Instruments Inc. 2013). All harvested roots showed a diameter <2 mm and were as such considered fine roots (Pérez‐Harguindeguy et al., 2013). Root length (m) and total root surface area (cm^2^) were determined with WinRhizo® software (Regent Instruments Inc. 2013). Subsequently, samples were dried (70°C, 72 h), and weights were added to the remaining root weights.

Fitness in our study is represented by total biomass, that is, the sum of aboveground and belowground biomass. Reproductive success, which is normally used to define the fitness of a genotype compared with all genotypes in its population, was not suited as a fitness measure in our study, as we terminated the experiment after 8 weeks, which was too short for some species to enter their generative stage. For example, at the time of harvest, 100% of individuals flowered in the species *Avena sterilis*, *Bromus secalinus*, and *Lolium remotum*, but only 53% in *Epilobium anagallidifolium* and 60% in *Lupinus angustifolius*, whereas no individual of *Hordeum murinum* or *Epilobium fleischeri* had produced flowers. The last two species reached their flowering time only after an average of 80 and 200 days, respectively (observation from another study with the same experimental setup). We thus decided to use biomass as an indicator of fitness, as suggested by Younginger et al. (2017).

### 2.4 Data and statistical analyses

We first determined if the measured traits responded plastically to our treatments, by conducting several ANOVA analyses: (a) for all species combined, (b) separately for each group of endangered, nonendangered, and invasive species, and (c) for each species separately (Table 1), we tested the effect of treatment on fitness (i.e., total biomass) and on every other measured trait, each in a separate analysis. To specifically address the effects of variation in either N or P availability on trait plasticity, we split the treatments into two groups: one group with N‐limited and balanced nutrient conditions and one group with P‐limited and balanced nutrient conditions, so that for each group, treatment term comprised two levels (N limited and balanced and P limited and balanced, respectively). Species (17 levels) and status (endangered, nonendangered, and invasive, 3 levels) were included as random factors where applicable, as was the date of harvest (10 levels). The harvest days of the 17 species were distributed over a total of 10 different dates, depending on the starting date of the experiment for each species. On each of these dates, all individuals of a specific species were harvested. As we already included species as random factors in our analyses, functional group (3 levels, nonlegume forbs, grasses, and legumes) as an additional random factor was not included in the models. We did this as we considered the inclusion of possible differences, such as differences in growth patterns, sufficiently incorporated by species as random factors. The analyses which involved *L. angustifolius* potsize were also included as random factors (2 levels, 500 and 3000 mL pots). As such, the analyses combining all species included date of harvest, status, and potsize as random factors, the analyses for each group separately included species and date of harvest as random factors. A significant treatment term in the analyses of fitness indicated that the applied nutrient manipulation effectively influenced biomass production. A significant treatment term in the analyses of the remaining traits indicated a plastic response of this trait to variation in nutrient availability. Data were beforehand tested on the normal distribution of residuals and homogeneity of variance and were either log or square‐root transformed if applicable.

We then analyzed if plasticity in traits was costly for fitness (i.e., total biomass, DeWitt et al., 1998, van Kleunen et al., 2000). This was done only for those traits that responded plastically to the treatments, as a result of the analyses mentioned above, and again (a) for all species combined, (b) separately for each group of endangered, nonendangered, and invasive species, and (c) for each species separately. We used regression analysis with mean fitness in each treatment as the response variable. The independent variables were the aforementioned plastically responding traits in the same treatment as mean fitness and a measure of plasticity across treatments for the same trait, following the approach of Scheiner and Berrigan (1998), Steinger et al. (2003), and Caruso et al. (2006). For example, mean fitness in the balanced treatment was regressed on a trait obtained from plants in the balanced treatment and a measure of the plasticity of the same trait obtained from plants across the balanced and the N‐limited treatment (or P‐limited treatment, respectively). As such, our model, *W ~ X + plX* used *W* as the relativized fitness, *X* as the trait values in this environment, and *plX* as a measure of the plasticity of that trait (e.g., *W*_Balanced_ *~ X*_Balanced_ *+ plX*_Balanced/N limited_ or *W*_N limited_ *~ X*_N limited_ *+ plX*_N limited/Balanced_). The measure of plasticity was calculated as the absolute difference in trait values between the nutrient treatments. To allow direct comparisons between models, fitness was relativized by dividing by mean fitness, and the independent variables were standardized prior to analysis (mean = 0, SD = 1). A significant negative regression coefficient for the plasticity term indicated that plasticity was costly in this environment (for conceptual graphs and interpretation of the direction of the regression coefficients for all statistical analyses, see Figure S1).

We then used two complementary regression approaches to determine if plasticity (for all plastic traits) or if lack of plasticity (for all nonplastic traits) in traits was adaptive, maladaptive, or neutral. The first approach considered only the nutrient‐limited treatments, that is, within N‐limited and within P‐limited treatments, respectively, and is the so‐called “within‐environment phenotypic selection analysis for adaptive plasticity” after van Kleunen and Fischer (2005). We did this (a) for all species combined, (b) separately for each group of endangered, nonendangered, and invasive species, and (c) for each species separately. For this, we regressed the relativized fitness on its standardized trait (see Caruso et al., 2006; *within environment*, either *W*_N limited_ *~ X*_N limited_ or *W*_P limited_ *~ X*_P limited_). For the plastic traits, a significant positive regression coefficient of the trait indicated that plasticity for that trait was adaptive. Conversely, a significant negative regression coefficient of the trait indicated that plasticity for that trait was maladaptive, whereas a nonsignificant regression coefficient indicated neutrality. A significant regression coefficient for a nonplastic trait indicated that the lack of plasticity in this trait is maladaptive. Last, a nonsignificant regression coefficient indicated that the lack of plasticity in this trait is adaptively neutral.

The second approach focused on regression analyses *across environments,* that is, across one of the nutrient‐limited treatments and the balanced nutrient treatment, respectively. These environments encompassed N‐limited and balanced and P‐limited and balanced treatments, respectively, and the approach is called “across‐environment genotypic selection analysis for adaptive plasticity” (Van Kleunen & Fischer, 2001). The relativized fitness across treatments (i.e., N limited/Balanced or P limited/Balanced) was regressed on its standardized trait across treatments and a standardized measure of plasticity. The measure of plasticity was calculated as the absolute difference in trait values between the nutrient treatments, that is, either *W*_N limited/Balanced_ *~ X*_N limited/Balanced_ *+ plX*_N limited/Balanced_ or *W*_P limited/Balanced_ *~ X*_P limited/Balanced_ *+ plX*_P limited/Balanced_. We did this for all measured traits, that is, for all plastic and nonplastic traits and (a) for all species combined, (b) separately for each group of endangered, nonendangered, and invasive species, and c) for each species separately. For the plastic traits, a significant and positive regression coefficient for the plasticity term indicated that plasticity was adaptive across both environments (significant and negative: plasticity was maladaptive, nonsignificant: plasticity was neutral). For nonplastic traits, a significant positive regression coefficient for the plasticity term indicated that a lack of plasticity in this trait is maladaptive. A significant and negative regression coefficient for the plasticity term indicated that a lack of plasticity in this trait is adaptive. Last, a nonsignificant regression coefficient indicated that a lack of plasticity is adaptively neutral.

## 3 RESULTS

### 3.1 Effects of nutrient availability on fitness and plasticity in traits

**FIGURE 1:**
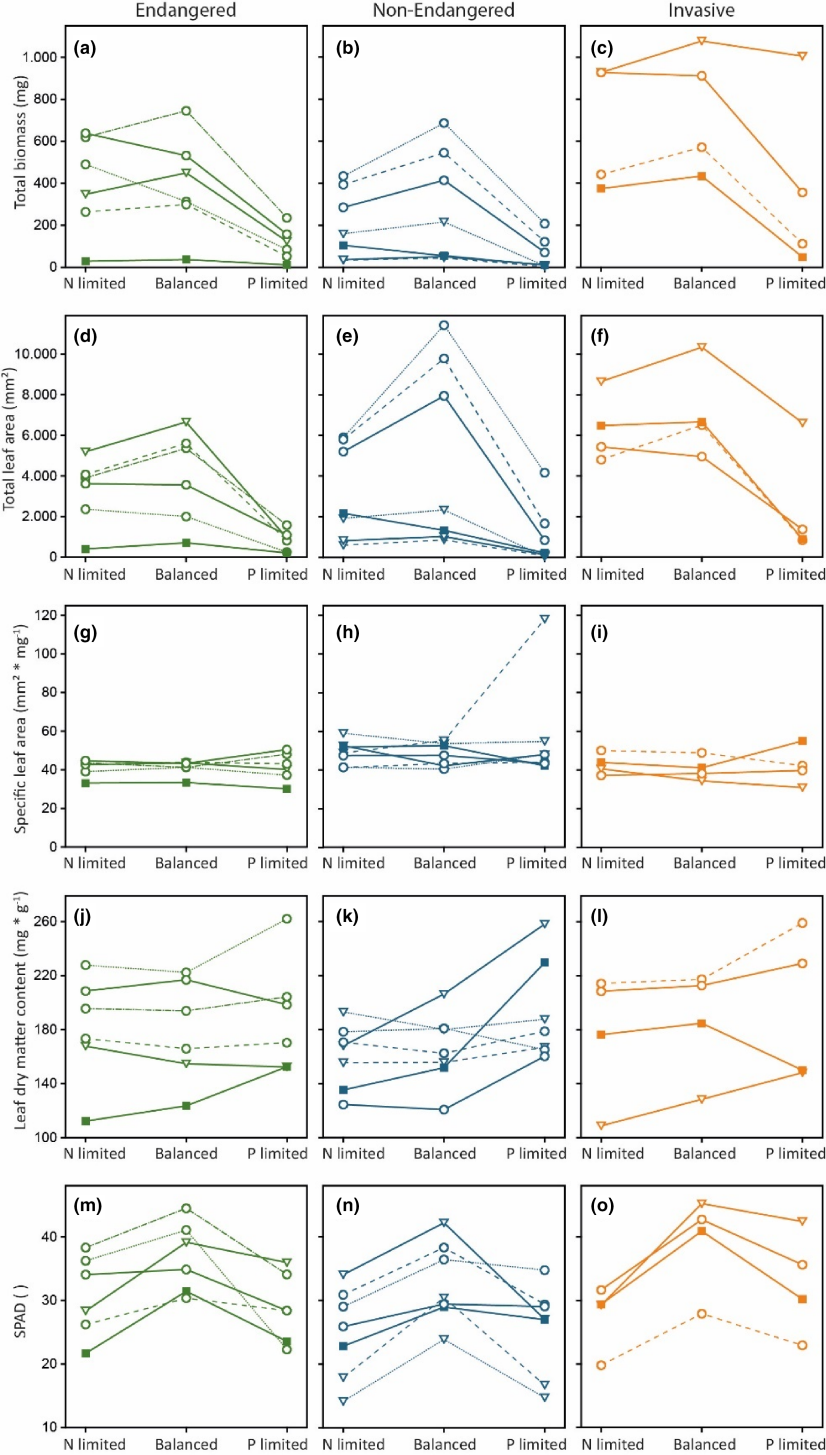
Mean response of fitness (total biomass, a–c), Leaf area (d–f), Specific leaf area (SLA, g–i), Leaf dry matter content (LDMC, j–l), and SPAD (m–o) of endangered (left graphs, green), nonendangered (middle graphs, blue), and invasive (right graphs, orange) species to N limitation, balanced nutrient supply, and P limitation. Endangered species: *Epilobium fleischeri*: filled square, solid line; *Bromus secalinus*: circle, solid line; *Bromus squarrosus*: circle, dashed line; *Lolium remotum*: circle, dotted line; *Lolium temulentum*: circle, dashed‐dotted line; *Trifolium subterraneum*: triangle, solid line. Nonendangered species: *Epilobium anagallidifolium*: filled square: solid line; *Bromus hordeaceus*: circle, solid line; *Bromus japonicus*: circle, dashed line; *Hordeum murinum*: circle, dotted line; *Medicago lupulina*: triangle, solid line; *Trifolium arvense*: triangle, dashed line; *Trifolium dubium*: triangle, dotted line. Invasive species: *Avena sterilis*: circle, solid line; *Epilobium ciliatum*: filled square, solid line; *Hordeum jubatum*: circle, dotted line; *Lupinus angustifolius*: triangle, solid line.

N limitation significantly affected biomass production in nonendangered species, with less biomass produced in the N‐limited treatment than under balanced nutrient supply (Table 2 and Figure 1b, but see higher biomass produced under N limitation than under balanced nutrient supply in *E. anagallidifolium*). Fitness of endangered or invasive species, as well as of all species combined, did not show a significant effect of N limitation (Table 2). Under P limitation fitness across all species as well as of the three groups separately was lower than under balanced nutrient supply (Table 2, see Figure S3 for fitness and trait responses across all species). The separate analyses for each species revealed that variation in P availability affected total biomass in 16 out of 17 species, whereas biomass of 8 out of 17 was influenced by variation in N availability (Table S3).

**TABLE 2:** *F*‐values and significance levels for one‐way ANOVA testing the effects of treatment (N limited vs. balanced supply [upper part] or P limited vs. balanced supply [lower part]) on fitness (total biomass) and other traits across all species and for endangered, nonendangered, and invasive species separately.

	All species	Endangered	Nonendangered	Invasive
N limitation versus balanced nutrient supply				
Total biomass	2.75	0.003	5.16*	1.47
Leaf area	6.05*	0.14	2.17	2.95
SLA	0.92	0.14	0.14	1.86
LDMC	0.09	0.95	0.27	0.76
SPAD	85.24***	22.50***	35.16***	43.84***
RMR	6.23*	6.27*	2.49	0.11
Root length	6.66*	4.78*	1.17	0.65
SRL	6.57*	1.11	13.18***	1.74
Surface area	1.46	3.22	0.0003	0.06
PME activity	291.12***	63.00***	112.00***	81.23***
P limitation versus Balanced nutrient supply				
Total biomass	95.60***	81.45***	48.86***	23.22***
Leaf area	140.05***	115.4***	46.15**	72.42***
SLA	1.62	0.66	1.27	0.14
LDMC	8.33**	1.63	4.97*	1.16
SPAD	45.08***	27.90***	16.83***	10.35**
RMR	59.73***	30.13***	23.28***	11.31**
Root length	108.47***	42.74***	33.8**	69.96***
SRL	2.69	0.446	4.31*	1.11
Surface area	143.47***	37.53***	39.75***	82.59***
PME activity	34.39***	10.68**	21.68***	11.91***

*Note*: For results for each species separately, see Table S3. For species names in each group, see Table 1. Total biomass (mg), leaf area (mm^2^), SLA (mm^2^ mg^−1^), leaf dry matter content (LDMC) (mg g^−1^), SPAD (–), RMR (mg mg^−1^), root length (m), specific root length (SRL) (m mg^−1^), root surface area (cm^2^), PME activity (μmol pNpp g^−1^ h^−1^). All significant F‐values are in bold.

*p < .05, **p < .01, ***p < .001.

For morphological or physiological traits and for all species combined, leaf area, SPAD, RMR, root length, SRL, and PME activity responded plastically to N‐related nutrient availability. In the group of endangered species, four traits responded plastically (SPAD, RMR, root length, and PME activity), three in the group of nonendangered species (SPAD, SRL, and PME activities) and two in the group of invasive species (SPAD and PME activity, see Table 2). Analyzed for each species separately, PME activity and SPAD values responded significantly to variation of N (except SPAD of *B. secalinus* and *M. lupulina*). To variation of P, leaf area (in 17 species), root length (in 14 species), root surface area (in 14 species), and PME activity (in 13 species) responded plastically (see Table S3).

**FIGURE 2:**
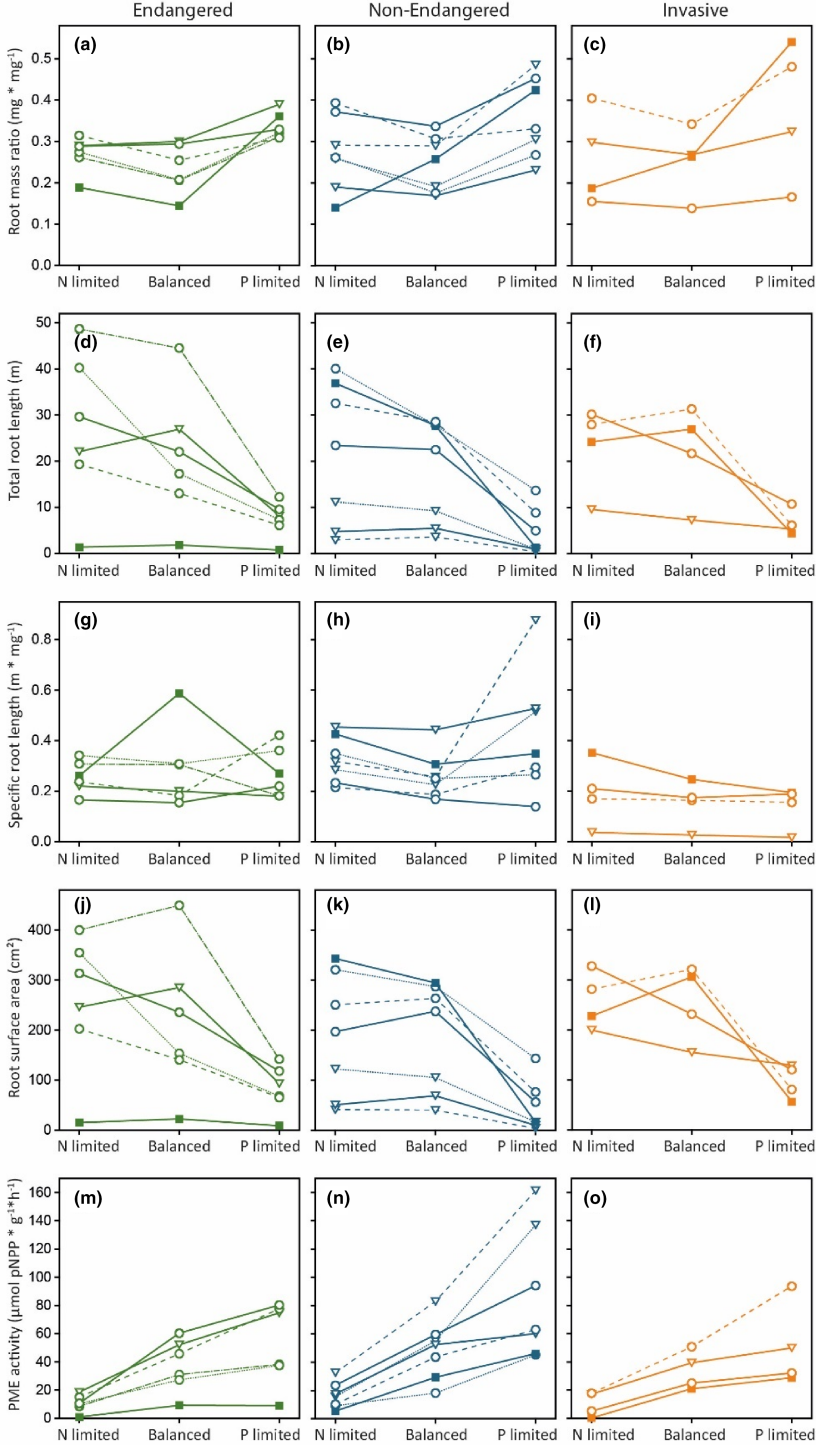
Mean response of root mass ratio (RMR, a–c), total root length (d–f), specific root length (SRL, g–i), root surface area (j–l), and PME activity (m–o) of endangered (left graphs, green), nonendangered (middle graphs, blue), and invasive (right graphs, orange) species to N limitation, balanced nutrient supply, and P limitation. For symbol and line configuration, see Figure 1.

SPAD values were lower in the N limited than in the balanced treatment, with the strongest difference for invasive species (29% lower in the N‐limited treatments, 14% for endangered, and 25% for nonendangered species, Figure 1m–o, see also Table S4 for mean and RSD for each species and trait, and Table S4 for mean and RSD for each group of species). Similarly, under N limitation, PME activity was lower than under balanced nutrient supply; 71% lower for endangered species, 65% for nonendangered species, and 71% for invasive species (Figure 2m–o). The remaining traits responded nonplastically to N‐related nutrient availability and only showed small differences between the treatments. For example, between the N‐limited and balanced nutrient treatments, SLA differed by 1.1% in the group of endangered species (1.7% for nonendangered and 5.4% for invasive species, Figure 1g–i). Similarly, leaf area and LDMC varied little (for leaf area: 13% for endangered and 12% for invasive species but 36% for nonendangered species (Figure 1d–f), for LDMC: 3.8% for endangered, 1.9% for nonendangered and 4.6% for invasive species (Figure 1j–l), respectively).

When P availability was varied against balanced nutrient supply, six traits responded plastically in the group of endangered species, eight in the group of nonendangered, and six in the group of invasive species (Table 2). The strongest difference between the P‐limited and the balanced nutrient treatments could be found for leaf area, with endangered species showing 78% lower leaf area under P limitation (75% in nonendangered and 67% in invasive species, Figure 1d–f). Similarly, invasive species showed a total root length that was 68% smaller under P limitation than under balanced nutrient supply (64% smaller in both endangered and nonendangered species, Figure 2d–f). SPAD values were on average 18% lower under P limitation compared with a balanced nutrient supply in endangered and nonendangered species and 16% in invasive species, respectively (Figure 1m–o). Similar to the variation of N availability, SLA did not respond plastically to the variation of P availability, irrespective of status (i.e., if a species was considered endangered, nonendangered, or invasive) or across all 17 species. LDMC only responded plastically in the group of nonendangered species, as did SRL (with 14% higher LDMC values under P limitation and 45% higher values for SRL, respectively, Figures 1k and 2h).

### 3.2 Costs of plasticity

Irrespective of treatment or status, high SPAD values positively affected fitness (Table 3). Investment into PME activity affected fitness differently in endangered and nonendangered species depending on nutrient treatment: under N limitation, PME activity negatively affected fitness in endangered species, but not under balanced nutrient supply, whereas in nonendangered species, fitness was negatively affected by PME activity under balanced nutrient supply, but not under N limitation. All species combined and endangered species separately showed that a high total root length was associated with a higher fitness both under N limited and balanced nutrient supply, respectively. Investment into high SRL leads to lower fitness under N limitation compared with balanced nutrient supply in invasive species. High leaf area contributed to higher fitness in several species when analyzed separately, for example, in *B. squarrosus*, *Lo. temulentum*, *T. subterraneum*, *B. hordeaceus*, and *H. murinum* (Table S6).

**TABLE 3:** Analysis of costs of plasticity in morphological and physiological traits across all species, and for endangered, nonendangered, and invasive species separately.

	Balanced		N limited		Balanced		N limited	
	*X* (Bal)	*plX* (Bal‐Nlim)	*X* (N lim)	*plX* (N lim‐Bal)	*X* (Bal)	*plX* (Bal‐N lim)	*X* (N lim)	*plX* (N lim‐Bal)
	All species				Endangered species			
Leaf area	0.493***	−0.066	0.495***	−0.138**				
SPAD	0.384***	−0.091	0.412***	0.129**	0.293**	−0.136*	0.391**	−0.088
RMR	0.042	−0.118*	0.067	−0.047	0.133	−0.119	0.116	0.065
Root length	0.277***	−0.057	0.301***	−0.038	0.404**	0.022	0.408**	−0.017
SRL	−0.298***	−0.105*	−0.288***	−0.095				
Surface area							0.423**	0.006
PME activity	−0.165*	−0.067	−0.220***	−0.049	−0.024	0.072	−0.299**	0.179
	Nonendangered species				Invasive species			
SPAD	1.265**	−0.334**	0.601**	0.001	0.212**	−0.067	0.189**	0.039
RMR								
Root length								
SRL	−0.289*	0.034	−0.225	−0.097			−0.230**	−0.061
Surface area								
PME activity	−0.405**	−0.129	−0.233	−0.210	0.122	−0.122	0.113	−0.131

	Balanced		P limited		Balanced		P limited	
	*X* (Bal)	*plX* (Bal‐P lim)	*X* (P lim)	*plX* (P lim‐Bal)	*X* (Bal)	*plX* (Bal‐P lim)	*X* (P lim)	*plX* (P lim‐Bal)
	All species				Endangered species			
Leaf area	0.547***	−0.177**	1.408***	−0.378***	0.367**	−0.156	0.627**	−0.203**
LDMC	0.109	−0.205***	−0.197	−0.273				
SPAD	0.397***	−0.132*	0.960***	−0.065	0.372**	−0.066	0.308*	0.157
RMR	0.053	−0.167**	−0.135	−0.256*	0.109	−0.149*	0.092	−0.181
Root length	0.261***	−0.077	0.357	−0.576***	0.329**	0.109	0.625**	0.102
SRL								
Surface area	0.375***	−0.131	0.963***	−0.405**	0.398**	0.033	0.554**	0.171**
PME activity	−0.123	−0.175**	−0.401	−0.216	0.101	−0.169*	−0.071	−0.164
	Nonendangered species				Invasive species			
Leaf area	0.848**	−0.001	1.096**	0.182*	0.114*	−0.226**	0.951**	−0.272**
LDMC	0.106	−0.459**	−0.342	−0.596**				
SPAD	0.579**	−0.292**	0.926**	−0.099	0.153*	−0.118	0.772**	−0.311**
RMR	0.089	−0.161	−0.052	−0.265	−0.015	−0.193**	−0.269	−0.231
Root length	0.749**	−0.047	1.329**	0.095	−0.068	−0.214**	−0.078	−0.854**
SRL	−0.167	−0.338*	−0.542*	0.103				
Surface area	0.754**	0.110	1.276**	0.064	0.010	−0.237**	0.564**	−0.383*
PME activity	−0.407**	−0.229	−1.039**	−0.324	0.081	−0.159*	−0.108	−0.184

*Note*: Costs were determined by regressing the mean fitness within one treatment (N limited, Balanced, or P limited) on the mean of a trait in this treatment and a measure of plasticity across two treatments (N limited/balanced [upper part] or P limited/balanced [lower part], respectively). Given are regression coefficients for the trait values (*X*) in the specific environment. A significant negative regression coefficient for the plasticity term (*plX*) indicates that it is costly (positive and significant or nonsignificant: noncostly). An empty field indicates that the respective trait was nonplastic. All significant F‐values are in bold.

*p < .05, **p < .01, ***p < .001.

We detected one significant negative regression coefficient for plasticity in SPAD in endangered and nonendangered species under balanced nutrient supply (*plX*: −0.316, *p*: .036 [endangered], *plX*: −0.334, *p*: .002 [nonendangered]), but not under N limitation (*plX*: −0.088, *p*: .14 [endangered], *plX*: 0.001, *p*: .98 [nonendangered]). This indicated that endangered and nonendangered species that exhibited high plasticity in SPAD had lower fitness under balanced nutrient supply, compared with their counterparts with high plasticity in the N‐limited treatments. As such, plasticity in SPAD incurred fitness costs in the favorable (balanced) nutrient treatment.

The comparison between balanced nutrient supply and P‐limited conditions showed that a high leaf area affected fitness positively, as did high SPAD values and a high root surface area, irrespective of treatment and whether a species was endangered, nonendangered, or invasive, respectively (see significant regression coefficients for trait values (*X*), Table 3). Fitness responded positively to high total root length under balanced conditions and under P limitation in endangered and nonendangered species (*X*: 0.329 and 0.625 [endangered], *X*: 0.749 and 1.329 [nonendangered], Table 3). The comparison of treatments (balanced vs. P limitation) regarding the effects of trait plasticity revealed costs in plasticity for the traits RMR and PME activity in the balanced treatment in endangered species, whereas these traits did not impose fitness costs in the P‐limited treatments. Similarly, plasticity in SPAD yielded lower fitness in nonendangered species under balanced conditions (*plX*: −0.292, *p*: .008), but not under P limitation (*plX*: −0.099, *p*: .576), whereas plasticity in LDMC imposed costs for fitness under both balanced and P‐limited conditions (*plX*: −0.459, *p*: <.001 [balanced], *plX*: −0.596, *p*: .002 [P limitation], respectively). Last and similar to endangered species, plasticity in RMR and PME activity imposed fitness costs under balanced conditions in invasive species but not under P limitation, whereas we found the reverse for SPAD. Invasive species that showed high plasticity in total root length and root surface area showed lower fitness under both balanced and P‐limited conditions.

### 3.3 Within‐environment analyses of plastic and nonplastic traits

Within‐environment analyses of *plastic traits* showed that plants with higher SPAD values had a higher fitness under N limitation as well as under P limitation, for all species analyzed and irrespective of their status as endangered, nonendangered, or invasive (regression coefficients: 0.368, 0.399, 0.382, and 0.211 (N limitation), 0.981, 0.377, 0.786, and 0.803 (P limitation), all significant, Table 4, upper part). Similarly, plants with a higher leaf area and higher root surface area showed higher fitness under P limitation (regression coefficients: 1.358, 0.604, 1.211, and 1.011 [leaf area], 0.943, 0.625, 1.300, and 0.800 [root surface area], all significant).

**TABLE 4:** Regression coefficients for within‐environment analyses of plastic and nonplastic traits within the N‐limited or the P‐limited treatment.

	Within environment: N limited trt			
	All species	Endangered	Nonendangered	Invasive
	*X* (N lim)	*X* (N lim)	*X* (N lim)	*X* (N lim)
Plastic traits				
Leaf area	0.473***			
LDMC				
SPAD	0.368***	0.399**	0.382**	0.211**
RMR	0.080	0.165*		
Root length	0.273***	0.387**		
SRL	−0.349***		−0.256*	−0.254**
Surface area				
PME activity	−0.211***	−0.068	−0.348**	0.067
Nonplastic traits				
Leaf area		0.143*	0.749**	0.176*
SLA	−0.259***	0.114	−0.356**	−0.227**
LDMC	0.153**	0.287**	0.116	−0.126
RMR			0.366**	−0.074
Root length			0.589**	−0.082
SRL		−0.028		−0.254**
Surface area	0.402***	0.406**	0.532**	0.108

	Within environment: P limited trt			
	All species X	Endangered X	Nonendangered X	Invasive X
	(P lim)	(P lim)	(P lim)	(P lim)
Plastic traits				
Leaf area	1.358***	0.604**	1.211**	1.011**
LDMC	−0.202		−0.279	−0.419*
SPAD	0.981	0.377**	0.786**	0.803**
RMR	−0.225	0.037	−0.175	−0.396*
Root length	0.353*	0.620**	1.286**	0.042
SRL			−0.571**	−0.739**
Surface area	0.943***	0.625**	1.300**	0.800**
PME activity	−0.428**	−0.054	−0.747**	−0.227
**Nonplastic traits**				
Leaf area				
SLA	−0.355**	0.176	−0.240	−0.663**
LDMC		0.143		−0.419*
RMR				
Root length				
SRL	−0.706***	−0.403**		−0.739**
Surface area				

*Note*: Analyses were run for all species as well as for endangered, nonendangered, and invasive species separately. Fitness in one treatment was regressed on each trait in this environment. Significant regression coefficients for the trait values (*X*) in the specific environment are in bold.

*p < .05, **p < .01, ***p < .001.

The remaining plastic traits in endangered species were either adaptive (N limitation: RMR, N and P limitation: total root length) or adaptively neutral (N limitation: PME activity, P limitation: PME activity, and RMR). Whereas endangered plants with higher RMR had significantly higher fitness under N limitation, variation in PME activity under N and P limitations, and RMR under P limitation had no effect on fitness. Plasticity in traits of nonendangered species was maladaptive for PME activity, both under P and N limitation. This suggests that lower PME activity both under N and P limitation caused higher fitness in these plants. Further, LDMC and RMR were adaptively neutral, as they had no effect on fitness under P limitation in nonendangered species. PME activity under N and P limitation and total root length under P limitation in invasive species were adaptively neutral, whereas plasticity in RMR was maladaptive under P limitation. Plants showing lower values in these traits had a higher fitness under P limitation.

A comparison on the species level (i.e., for the analyses for each species separately) showed that the effects of trait plasticity on fitness were less pronounced under N limitation than under the P limitation (see coefficients in Table S7). Under N limitation, two traits affected fitness positively and 3 traits negatively (i.e., positive: root length in *B. squarrosus* and *Lo. remotum* and leaf area in *T. subterraneum*, *B. hordeaceus*, *B. japonicus*, and *H. jubatum,* negative: PME in *B. secalinus* and *Lo. temulentum*, SRL in *B. hordeaceus,* and root length in *H. murinum*), all other effects of plastic traits were nonsignificant. Under the P limitation, five traits affected fitness positively and three traits negatively. These were leaf area, SPAD, root length, surface area, and PME activity. Negative effects on fitness under P limitation were detected for SPAD, RMR, and SRL.

Within‐environment analyses of *nonplastic traits* suggested that a lack of plasticity in traits of endangered, nonendangered, and invasive plants can be maladaptive and neutral. For all species groups, high leaf area exerted a positive effect on fitness under N limitation, but this trait was not plastic, and as such this lack in plasticity was maladaptive (Table 4, lower part). Similarly, endangered species that showed a high LDMC and root surface area and nonendangered species with low SLA and high RMR, total root length, and root surface area showed a higher fitness under N limitation; lack of plasticity in these traits was equally maladaptive. Last, under N limitation, low SLA, and low SRL were maladaptive in invasive species. A lack of plasticity in SLA and SRL in endangered species, LDMC in nonendangered species, and LDMC, RMR, and root surface area in invasive species were adaptively neutral.

Under P limitation, lack of plasticity in SLA and SRL was adaptive for all species and invasive species separately, respectively. A lack of plasticity in LDMC in endangered species and SLA in endangered and nonendangered species was adaptively neutral.

### 3.4 Across‐environment analyses of plastic and nonplastic traits

Across N limited and balanced nutrient supply, our results showed that the regression coefficients for the plastic traits SPAD, RMR, and total root length in endangered species exerted a positive significant effect on fitness, whereas the regression coefficient for PME activity was nonsignificant (Table 5). Across P‐limited and balanced nutrient supply, regression coefficients showed that high leaf area and high SPAD values positively affected fitness in endangered, nonendangered, and invasive plants; there were positive effects also from total root length and root surface area on endangered and nonendangered plants, see significant positive regression coefficients for the trait values (*X)* in Table 5. Contrary, high PME activity negatively affected fitness in endangered and nonendangered plants, as did high root surface area in invasive species and high RMR and SRL in both nonendangered and invasive species.

**TABLE 5:** Across‐environment analyses of adaptive plasticity (for plastic traits) and whether a lack of plasticity is adaptive, maladaptive, or neutral (for nonplastic traits).

	Across‐environment analyses: N limited versus balanced treatment							
	All species		Endangered		Nonendangered		Invasive	
	*X* (Nlim‐Bal)	*plX* (Nlim‐Bal)	*X* (Nlim‐Bal)	*plX* (Nlim‐Bal)	*X* (Nlim‐Bal)	*plX* (Nlim‐Bal)	*X* (Nlim‐Bal)	*plX* (Nlim‐Bal)
Plastic traits								
Leaf area	0.486***	−0.089*						
SPAD	0.348***	0.012	0.272**	−0.103*	0.522**	−0.213**	0.171**	0.010
RMR	0.044	−0.084*	0.115*	−0.021	−0.336**	−0.007		
Root length	0.273***	−0.048	0.379**	−0.010				
SRL	−0.303***	−0.099**					−0.233**	−0.073
Surface area								
PME activity	−0.019	−0.155***	−0.048	0.087	0.001	−0.359**	0.117*	−0.107
Nonplastic traits								
Leaf area			0.257**	−0.109*	0.797**	0.068	0.103	0.069
SLA	−0.230***	−0.109**	0.082	−0.029	−0.296**	−0.259**	−0.217**	0.039
LDMC	0.129**	−0.139***	0.207**	−0.114**	0.088	−0.357**	−0.105*	0.059
RMR					0.184*	0.050	−0.060	−0.077
Root length					0.622**	−0.014	−0.117*	−0.089*
SRL			−0.003	−0.029			−0.233**	−0.073
Surface area	0.361***	−0.037	0.389**	0.006	0.710**	−0.119	−0.047	−0.025

	Across‐environment analyses: P limited versus balanced treatment							
	All species		Endangered		Nonendangered		Invasive	
	*X* (Plim‐Bal)	*plX* (Plim‐Bal)	*X* (Plim‐Bal)	*plX* (Plim‐Bal)	*X* (Plim‐Bal)	*plX* (Plim‐Bal)	*X* (Plim‐Bal)	*plX* (Plim‐Bal)
Plastic traits								
Leaf area	0.844***	−0.104*	0.717**	−0.208	1.231**	−0.004	0.527**	−0.276*
LDMC	−0.047	−0.178**			−0.219	−0.467**		
SPAD	0.648***	0.029	0.568**	0.025	0.896**	−0.185	0.454**	−0.171**
RMR	−0.257***	−0.104	−0.163	−0.102	−0.247*	−0.009	−0.364**	0.029
Root length	0.549***	−0.129*	0.752**	0.016	1.156**	−0.112	0.115	−0.176*
SRL					−0.338*	−0.263		
Surface area	0.650***	−0.084	0.782**	−0.030	1.346**	0.048	−0.339**	−0.448**
PME activity	−0.483***	0.036	−0.275*	−0.049	−1.034**	−0.152	−0.183	−0.063
Nonplastic traits								
SLA	−0.295***	−0.088	−0.026	−0.042	−0.219	−0.361*	−0.340**	−0.091
LDMC			0.106	−0.087			−0.234**	−0.079
SRL	−0.363***	−0.142*	−0.188	−0.150			−0.297**	−0.079

*Note*: Analyses were run for all species, as well as for endangered, nonendangered, and invasive species separately (for analyses for each species separately, see Table S8). Fitness across two treatments (i.e., either N limited/Balanced or P limited/Balanced) was regressed on each trait (*X*) and a measure of plasticity (*plX*) across those treatments. Significant (*p* < .05) coefficients are in bold.

*p < .05, **p < .01, ***p < .001.

Plasticity for most traits was maladaptive, meaning high plasticity exerted a negative effect on fitness or adaptively neutral, meaning variation in these traits did not affect fitness. For N limitation versus balanced conditions, high variation in SPAD affected fitness negatively in endangered and nonendangered species, as did PME activity in nonendangered and invasive species. Across P‐limited and balanced nutrient conditions, the plasticity of all plastic traits was neutral for endangered and nonendangered species, with the exception of LDMC in nonendangered species, which indicated that variation in these traits was insignificant for the fitness of these species. In contrast, plasticity for four out of seven traits for invasive species was maladaptive: high variation in leaf area, SPAD, total root length, and root surface area resulted in lower fitness across P‐limited and balanced nutrient conditions.

Across‐environment analyses, that is, across N limited and balanced nutrient supply, of nonplastic traits on fitness revealed that a high leaf area and a high LDMC positively affected fitness in endangered species (regression coefficients for *X*: 0.257 and 0.207, see Table 5), and coefficients of plasticity revealed that lack of plasticity was adaptive for leaf area and LDMC (*plX*: −0.109 and − 0.114, respectively). For nonendangered species, high SLA negatively affected fitness (*X*: −0.296) and a lack of plasticity in this trait was adaptive (*plX*: −0.259), as less plasticity in this trait resulted in plants with higher fitness. In invasive plants, results suggest that lack of plasticity was adaptively neutral in all traits but total root length, for which it was adaptive (*plX*: −0.089). Across‐environment analyses of P‐limited and balanced nutrient supply showed that lack of plasticity in SLA was adaptive in nonendangered plants (*plX*: −0.361), whereas plasticity in all other nonplastic traits was adaptively neutral, irrespective of species status.

## 4 DISCUSSION

### 4.1 Trait plasticity in endangered, nonendangered, and invasive species

We expected traits from invasive species to be more plastic than those of nonendangered or endangered species and traits of endangered species to be the least plastic. This has not been confirmed by our results. When we varied N availability, we detected four plant traits responding plastically across all species (see Table 2), four in endangered species, three in nonendangered species, and two in invasive species. When P availability was varied, seven traits responded plastically across all species, six in endangered species, eight in nonendangered, and six in invasive species, respectively. We conclude from these results that (a) when N availability is varied, the sheer number of plastically responding traits is highest in endangered and lowest in invasive species, (b) when P availability is varied, the total number of plastically responding traits is similar between the three groups of species, (c) more traits responded plastically to variation of P than to variation of N, irrespective of species' status, and (d) two traits responded plastically in all groups of species under variation of N (SPAD and PME activity) and six under variation of P (leaf area, SPAD, RMR, root length, root surface area, and PME activity).

Our three first observations mentioned above contradict the notion that invasive species show higher trait plasticity than nonendangered or endangered species. This is in concordance with a recent experimental study by Zhang et al. (2022), who manipulated water, light, and nutrient availability, as well as with a meta‐analysis by Liu et al. (2017) investigating the responses of alien and native species to elevated CO_2_, temperature, and N availability. Contrary, another meta‐analysis reported that invasive species indeed showed higher trait plasticity than noninvasive species (Davidson et al., 2011). The controversial conclusions of these studies may arise from different plant traits under study or dissimilar trait responses toward different resources (Colautti et al., 2017).

The meta‐analysis by Davidson et al. (2011) also showed higher adaptive fitness response of native species compared with invasive species. Contrary, our study showed no differences in trait adaptability between the three groups of species, which contradicts our second initial hypothesis. Whereas most of our analyzed trait plasticity was neutral toward fitness, we could identify plasticity in three traits being similarly adaptive across all species groups: SPAD (adaptive to N and P limitation), root surface area, and leaf area (adaptive to P limitation). More generally, we conclude that differences in trait plasticity and their effects on fitness were found between the type of nutrient that was varied (N and P), but they were similar between endangered, nonendangered, and invasive plant species.

### 4.2 Costs of plasticity

Almost all of our analyses for costs of plasticity were nonsignificant when N was varied, the only exception was a significantly negative regression coefficient for plasticity in SPAD in nonendangered species. Under variation of P, plasticity in RMR and PME activity was costly for fitness in endangered species under balanced conditions, but not under P‐limited conditions (Table 3). Plasticity in LDMC was costly for fitness in nonendangered species under balanced and P‐limited treatments, as was plasticity in root length and root surface area under the same conditions for invasive species. Various studies reported only little costs of plasticity (Auld et al., 2010; Caruso et al., 2006) and suggest that costs of plasticity may be more apparent in stressful environments (Hendry, 2016). Certainly, the nutrient conditions we created were quite stressful in both the N‐ and P‐limited treatments, which is apparent from the decrease in biomass production in comparison with the balanced nutrient supply (Figure 1a–c). However, we could only detect costs of plasticity under variation of P, not under variation of N (for all species combined, the three species groups, and also for each species separately), which suggests that costs of plasticity differ in respect to the type of limiting nutrient, that is, if N or P is in limitation, and not just that nutrients are limiting. Similarly, the study by Caruso et al. (2006) exposed two species of *Lobelia* (*L. cardinalis* and *L. siphilitica*) to dry and wet soil conditions and measured fitness‐related traits (biomass) and photosynthesis‐related traits. They did not detect any costs of plasticity under dry and wet conditions and concluded that costs of plasticity may be differently affected by the total amount of water or the timing of water accessibility.

Further, our results show that trait plasticity in response to P variation negatively affected fitness under “benign” conditions (i.e., under balanced nutrient supply) but was neutral under P limitation (i.e., compare negative coefficients of plasticity for SPAD in nonendangered species or PME activity in endangered and invasive species, Table 3). This indicates that showing plasticity toward stressful conditions (here P limitation) can come with costs in less stressful conditions (here balanced nutrient supply). For example, plasticity in PME activity toward variation in P in endangered and invasive species negatively affected fitness under balanced nutrient supply, probably because the production of this enzyme caused a loss of N otherwise available for carbon assimilation (Olde Venterink & Güsewell, 2010). Surprisingly, plasticity in PME activity was adaptively neutral within and across balanced and P‐limited treatments, irrespective of species' status (maladaptive for nonendangered species, see Tables 4 and 5). High PME activity has been suggested to contribute to competitive strength in *Agrostis capillaris* and is generally seen as an important adaptation under P limitation (Olde Venterink, 2011b; Olde Venterink & Güsewell, 2010). Our results do not contradict those findings but suggest that plasticity in this trait does not contribute to fitness, neither within P‐limited conditions nor across a gradient from P limitation to balanced nutrient supply, at least in our experiment that excluded the effects of competition.

### 4.3 Trait plasticity within and across nutrient environments

A trait responding plastically to fluctuating environmental conditions is not automatically adaptive (Richards et al., 2006). Similarly, plasticity may be of adaptive value in one environment, but that does not necessarily mean it is also adaptive across multiple environments (Hendry, 2016). For example, in the study by Caruso et al. (2006), plasticity in photosynthetic capacity (*A*_max_) was adaptively neutral in both the wet and dry treatments for *Lobelia siphilitica*. For *L. cardinalis* however, plasticity in the same trait was adaptively neutral in the wet treatment and maladaptive in the dry treatment. Our results show that under nutrient limitation (i.e., under N or P limitation) plastic traits were in parts adaptive, in parts maladaptive, and in parts neutral (*within*‐*environment analyses*, Table 4). Similarly, we detected that a lack of plasticity in nonplastic traits was adaptive, maladaptive, and adaptively neutral (Table 4). The results *across environments* (i.e., across N limitation and balanced nutrient supply, and across P limitation and balanced nutrient supply, Table 5) show that the majority of the trait plasticity was adaptively neutral, and in a few traits, it was maladaptive.

In his symposium article about on the role of plasticity in eco‐evolutionary dynamics, Hendry (2016) remarks that instead of solely identifying plasticity as adaptive, maladaptive, and neutral, it would be more informative to identify the conditions under which plasticity has its greatest (or least) adaptive value. Our analyses showed that within one environment, that is, only under N limitation or only under P limitation, plasticity in SPAD was adaptive (both N and P limitation), and leaf area and root surface area were adaptive under P limitation. White et al. (2013) highlight root surface area as important for the acquisition of mineral elements, especially for nitrate uptake, as it is delivered to roots predominately via mass flow, whereas high SRL is seen as beneficial for the uptake of P. Similarly, P limitation decreased leaf area in a study by Colomb et al. (2000) and reduced the total amount of absorbed PAR by up to 10%. Our study adds to this knowledge as we show that plasticity in both root surface area and leaf area contributes to fitness also under P limitation. As plasticity in SPAD was also adaptive under P limitation, there might be a mechanistic link between plasticity in leaf area and SPAD and their effects on fitness, but this needs to be investigated in another study.

Other traits that we measured were identified as plastic but showed nonconsistent adaptive value across the species groups. However, across different environments, plasticity in most traits was neutral or nonconsistent across species groups. In this context, one has to consider that the applied nutrient treatments may have affected the species of our experiment in different ways and that the species groups were unbalanced in terms of species number (6 endangered, 7 nonendangered, and 4 invasive species). For the latter, species number was included as a random effect in the analysis if possible and results obtained from analyses for each species separately did not deviate greatly from those for the species groups. For the first, what may have been severely nutrient limiting for one species, may have been less critical for another species, which in turn may have affected trait plasticity differently. This of course applies to all studies with similar study designs, and it is one of the reasons why multiple studies are needed to confirm the results at hand. Nevertheless, to our knowledge, this is the first study to identify plasticity in a set of different traits with an adaptive value under both N and P limitation and adaptive neutrality along a gradient of P for a set of different species.

## 5 SUMMARY

In summary, we found plastic trait responses to variation in N and P, with more traits responding plastically to P variation than to N variation and with only a few differences between the status of the plant species (i.e., whether they were endangered, nonendangered, or invasive). Costs of plasticity could only be detected when P was varied, with negative effects of plasticity on the “benign” end of the gradient, that is, under balanced nutrient supply. Most of our analyzed trait plasticity was neutral toward fitness, with plasticity in three traits being similarly adaptive across all species groups: SPAD (adaptive to N and P limitation), root surface area, and leaf area (adaptive to P limitation). More generally, we conclude that differences in trait plasticity and their effects on fitness were found between the type of nutrient that was varied (N and P), but they were similar between endangered, nonendangered, and invasive plant species. Under nutrient limitation, endangered, nonendangered, and invasive species of this study were similarly constrained, and high‐trait plasticity did not contribute to fitness. This may change if nutrient availability increases, with consequences on species abundances: in their recent study, Wassen et al. (2021) point out N enrichment as a major cause of species loss in European herbaceous ecosystems (Bobbink et al., 2010; Stevens, 2016). Species pools of endangered species are largest under low P availability, and the perspective of lowering N depositions as foreseen by European legislation (EEA, 2019; Schulte‐Uebbing & de Vries, 2021) will result in an increase of P availabilities relative to N, if P is not reduced simultaneously.

## AUTHOR CONTRIBUTIONS

**Vanessa Minden:** Conceptualization (lead); formal analysis (lead); funding acquisition (lead); writing – original draft (lead). **Koen Verhoeven:** Conceptualization (supporting); methodology (supporting); writing – review and editing (equal). **Harry Olde Venterink:** Conceptualization (supporting); funding acquisition (supporting); writing – review and editing (equal).

## CONFLICT OF INTEREST STATEMENT

The authors have no conflict of interest to declare.

## Supporting information


Appendix S1

## Data Availability

Data are publicly available through the Dryad repository (https://doi.org/10.5061/dryad.6m905qg4v).
