# Supplementary material for: Adaptive plasticity and fitness costs of endangered, nonendangered, and invasive plants in response to variation in nitrogen and phosphorus availabilities
Source: Ecol Evol. 2023 May 13;13(5):e10075. doi: 10.1002/ece3.10075 (PMC10182570; doi:10.1002/ece3.10075)
Supplement: Supplementary file 1 — Appendix S1 [file ECE3-13-e10075-s001.docx]

Electronic Supplemental Material: Online Resource 1 27th July 2022

Adaptive plasticity and fitness costs of endangered, non-endangered and invasive plants in response to variation in nitrogen and phosphorus availabilities

Vanessa Minden^1,*^, Koen Verhoeven^2^, Harry Olde Venterink^1^

^1^Department of Biology, Vrije Universiteit Brussel (VUB), Pleinlaan 2, 1050 Brussel, Belgium

^2^Terrestrial Ecology Department, Netherlands Institute of Ecology (NIOO-KNAW), Droevendaalsesteeg 10, 6708 PB Wageningen, The Netherlands.

Corresponding author: Vanessa Minden, Department of Biology, Ecology and Biodiversity, Vrije Universiteit Brussel, Brussels, Belgium. +32 2 629 3423, email: vanessa.minden@vub.be

**Supplementary Table S1**: Compilation of information on the study species in the groups of endangered, non-endangered and invasive species, with information on their Red List Categories and data on their native and introduced ranges, respectively. ^1^Royal Botanic Gardens Kiew (2021), ^2^IUCN (2021), ^3^Umweltbundesamt AT (2021), ^4^USDA-ARS (2016), ^5^Info Flora (2021), ^6^BiolFlor (2021), ^7^German Red List Centre (2021), ^8^Oberdorfer (2001), ^9^IUCN Red List (2021), ^10^Flora Web (2021), ^11^CABI (2021)

| Species name | | | Native to | Introduced to | Red List Category |
| --- | --- | --- | --- | --- | --- |
| **ENDANGERED SPECIES** | | | | | |
|  | Onagraceae | | |  |  |
| *Epilobium fleischeri* | | | Alps: Austria, France Germany, Switzerland, Italy ^1^ | no-where | Least Concern: Switzerland, Threatened: Austria, Critically endangered: Austria, Germany ^2,3^ |
|  | Fabaceae | | | | |
| *Trifolium subterraneum* | | | Europe to Kaukasus, Macaronesia to Iran ^1^ | Argentina, parts of Australia, Belarus, Brazil, Cape Province, Chile, Czech Republic, India, Japan, New Zealand, Tasmania, Uruguay, parts of the USA ^1^ | Vulnerable: Ireland, The Netherlands; Endangered: Hungary; Extinct: Malta ^2^ |
|  | Poaceae | | |  |  |
| *Bromus secalinus* | | | Europe (East, Central and Southwest), Iran, Siberia, North China ^1, 4^ | Algeria, Azores, Baltic States, parts of Canada, Canary Islands, Czech Republic, Denmark, Finland, Great Britain, Japan, Kamchatka, Korea, Norway, Palestine, Poland, parts of Russia, parts of South America, Sweden, Taiwan, parts of the USA ^1^ | Near Threatened: United Kingdom; Threatened: Germany; Critically Threatened: Austria, Czech Republic; Vulnerable: Switzerland; Endangered: Hungary, Sweden, Switzerland; Critically Endangered: Estonia ^2, 5^ |
| *Bromus squarrosus* | | | Central Europe to Mongolia, Mediterranean to Iran ^1^ | Baltic States, parts of Canada, Canary Islands, Chile, Manitoba, parts of Russia, parts of the USA ^1^ | Least concern: Switzerland; Critically Threatened: Austria, Czech Republic, Germany ^2, 3^ |
| *Lolium remotum* | | | North Pakistan to West Himalaya, archaeophyte in Europe ^1, 6^ | Afghanistan, Algeria, Argentina, Baltic States, Belarus, Canary Islands, Czech Republic, Finland, France, Great Britain, Hungary, Ireland, Irkutsk, Italy, Morocco, Newfoundland, Ontario, parts of Russia, Portugal, Sicilia, Spain, Ukraine, Xinjiang ^1^ | Critically endangered: Estonia, Switzerland; Regionally extinct: Sweden, The Netherlands; Extinct: Austria, Belgium, Germany, Hungary ^1, 2, 5^ |
| *Lolium temulentum* | | | Macaronesia, North Africa, Temperate Eurasia ^1^ | Argentina, Argentina, parts of Australia, Bangladesh, Bolivia, Brazil, parts of Canada, Cape Provinces, Chile, Colombia, Cuba, Himalaya, Ecuador, Eritrea, Ethiopia, Finland, Guatemala, Japan, Java, Kenya, KwaZulu-Natal, Lesotho, Manitoba, Mauritius, Mexico, Nepal, New Zealand, Norway, Panama, Peru, Philippines, Réunion, Switzerland, Tanzania, Tasmania, United Kingdom, Uruguay, parts of USA, Venezuela, Zimbabwe ^1^ | Endangered: France, Ireland; Critically endangered: Austria, Estonia, United Kingdom, Switzerland; Regionally extinct: The Netherlands; Extinct: Germany, Hungary, Java, Sweden ^1, 2, 3, 5, 7^ |
|  | | | | | |
|  | | | | | |
|  | | | | | |
|  | | | | | |
|  | | | | | |
|  | | | | | |
|  | | | | | |
|  | | | | | |
| **Supplementary Table S1** - *continued* | | | | | |
| Species name | | | Native to | Introduced to | Red List Category |
| **NON-ENDANGERED SPECIES** | | | | | |
|  | Onagraceae | | |  |  |
| *Epilobium anagallidifolium* | | | Subarctic and Temperate Northern Hemisphere ^1,8^ | none | Least Concern: United Kingdom, Switzerland; Threatened: Germany (Baden-Wuerttemberg), Czech Republic ^2, 5, 7,^ |
|  | | Fabaceae | | | |
| *Medicago lupulina* | | | Macaronesia, Europe to Caucasus, North and Northeast Tropical Africa to Arabian Peninsula, Indian Subcontinent to China ^1^ | Altay, Amur, Argentina, parts of Australia, Bahamas, Bermuda, Bolivia, Brazil, parts of Canada, Cape Provinces, Chile, Colombia, Cuba, Dominican Republic, Ecuador, Guatemala, Haiti, Iceland, Irkutsk, Japan, Kazakhstan, Kenya, Korea, KwaZulu-Natal, Mauritius, Mexico, New Zealand, Oman, Peru, Philippines, Réunion, parts of Russia, Tanzania, Tasmania, Turkmenistan, Uruguay, parts of the USA ^1^ | Least concern: Estonia, Germany, Ireland, Switzerland, The Netherlands, United Kingdom  ^2,5 7, 9^ |
| *Trifolium arvense* | | | Macaronesia, Europe to Central Asia and Iran, North Africa to Northeast Tropical Africa ^1^ | Altay, Amur, Argentina, parts of Australia, parts of Canada, Cape Provinces, Chile, Colombia, Haiti, Japan, New Zealand, parts of Russia, Sri Lanka, Uruguay, parts of the USA ^1^ | Least concern: Austria, Estonia Germany, Ireland, Switzerland, United Kingdom; Near threatened: Hungary  ^2, 5, 7,^ |
| *Trifolium dubium* | | | Macaronesia, Europe to Mediterranean and Caucasus ^1^ | Argentina, parts of Australia, Bolivia, Brazil, parts of Canada, Cape Provinces, Chile, China, Costa Rica, East Himalaya, Ecuador, Finland, Haiti, India, Jamaica, Japan, Java, New Guinea, New Zealand, Nicaragua, Pakistan, Panama, Peru, Réunion, parts of Russia, Sri Lanka, Taiwan, Tanzania, Tasmania, Uruguay, parts of the USA, Venezuela ^1^ | Least concern: Germany, Ireland, Switzerland, The Netherlands, United Kingdom ^2^ |
|  | Poaceae | | |  |  |
| *Bromus hordeaceus* | | | Macaronesia, Mediterranean, Europe to Russia and Himalaya ^1, 11^ | Argentina, parts of Australia, Brazil, parts of Canada, Cape Provinces, Chile, Falkland Islands, Greenland, Iceland, Japan, Korea, Mexico, New Zealand, parts of Russia, Taiwan, Tasmania, parts of the USA, Uruguay ^1^ | Least concern: Estonia, Germany, Ireland, Switzerland, The Netherlands, United Kingdom ^2, 7^ |
| *Bromus japonicus* | | | Europe (East, Central, Southeast and Southwest), Northern Africa (Egypt), Eurasia ^1,11^ | Australasia, North America, Argentina ^1^ | Near threatened: Switzerland, Czech Republic ^2^ |
| *Hordeum murinum* | | | Macaronesia, Europe, Mediterranean to Central Asia and West Himalaya ^1^ | Argentina, parts of Australia, Belarus, Bolivia, Brazil, parts of Canada, Cape Provinces, Chile, Denmark, Easter Islands, Falkland Islands, Georgia, Guatemala, Ireland, Japan, Korea, KwaZulu-Natal, Lesotho, Mexican Pacific Islands, Mexico, New Zealand, Norway, Peru, parts of Russia, Sweden, Tanzania, Tasmania, Uruguay, parts of the USA ^1^ | Least concern: Germany Ireland, Switzerland, The Netherlands, United Kingdom ^2, 5 7, 9^ |
|  | | |  |  |  |
|  | | |  |  |  |
|  | | |  |  |  |
|  | | |  |  |  |
|  | | |  |  |  |
|  | | |  |  |  |
| **Supplementary Table S1** - *continued* | | | |  |  |
| Species name | | | Native to | Introduced to | Red List Category |
| **INVASIVE SPECIES** | | | | | |
|  | Onagraceae | | |  |  |
| *Epilobium ciliatum* | | | South Siberia to Temperate East Asia, North America to Guatemala, South America ^1, 11^ | Antipodean Islands, Bulgaria, Czech Republic, Finland, Germany, Great Britain, Greece, Hawaii, Ireland, Malaysia, New Zealand, parts of Russia ^1, 11^ | Least concern ^9^ |
|  | Fabaceae | | | | |
| *Lupinus angustifolius* | | | Mediterranean ^1^ | Algeria, Argentina, parts of Australia, Austria, Azores, Baltic States, Belarus, Canary Islands, Cape Provinces, Chile, Czech Republic, Dominican Republic, Guatemala, Hungary, India, Jamaica, Kirgizstan, Korea, Madeira, Mongolia, New Zealand, Poland, Romania, parts of Russia, Rwanda, Switzerland, Ukraine, Uruguay, parts of the USA ^1^ | Germany: establishing occurrences ^10^ |
|  | Poaceae | | |  |  |
| *Avena sterilis* | | | Canary Islands, Mediterranean to West Himalaya and Kenya ^1^ | Argentina, parts of Australia, Austria, Azores, Belgium, Bolivia, Brazil, Cape Provinces, parts of Canada, Chile, China, Costa Rica, Czech Republic, Ecuador, Germany, Great Britain, Hungary, Madeira, New Zealand, parts of the USA, Peru, Réunion, Russia, Sri Lanka, Sweden, Switzerland, Tasmania, Uruguay, Venezuela, Western Sahara ^1, 11^ | Least concern ^9^ |
| *Hordeum jubatum* | | | North America, Siberia to Caucasus and Northeast China ^1^ | Argentina, Austria, Belgium, Bosnia-Herzegovina, Chile, Croatia, Czech Republic, Denmark, Falkland Islands, Finland, Germany, Italy, Korea, Kosovo, Labrador, Lesotho, Montenegro, New Zealand, Norway, parts of Russia, Slovenia, Sweden, Switzerland, The Netherlands, Ukraine, United Kingdom ^1^ | Least concern ^9^ |

BiolFlor. 2021. The Database BiolFlor on biological and ecological traits of the flora of Germany. Halle, Germany.

CABI. 2021. Invasive Species Compendium.

Flora Web. 2021. Data and Information on wild plants in Germany. Bundesamt für Naturschutz (BfN), Bonn, Germany.

German Red List Centre. 2021. Cologne, Germany.

Info Flora. 2021. The National Data and Information Center on the Swiss Flora.

IUCN. 2021. International Union for Conservation of Nature - National Red List.

IUCN Red List. 2021. The IUCN Red List of Threatened Species.

Oberdorfer, E. 2001. Pflanzensoziologische Exkursionsflora für Deutschland und angrenzende Gebiete. Eugen Ulmer, Stuttgart, Germany.

Royal Botanic Gardens Kiew. 2021. Plants of the world online.

Umweltbundesamt AT. 2021. Rote Listen gefährdeter Biotoptypen und Arten. Wien, Austria.

USDA-ARS. 2016. Germplasm Resources Information Network (GRIN). National Plant Germplasm System. Online Database. . National Germplasm Resources Laboratory, Beltsville, Maryland, USA.

**Supplementary Table S2:** Upper part: Total amount of N and P given to each plant individual during the course of the experiment and concentration of compound (either NaNO_3_ or NaH_2_PO_4_.2H_2_O) per litre nutrient solution.

Lower part: Total amount of macro- and micronutrients given to each plant individual during the course of the experiment, molar masses of compounds and elements and concentration of each compound weighed in per litre nutrient solution.

|  | Nitrogen (NaNO_3,_ molar mass 84.99 g/mol, molar mass N 14.01 g/mol) | | Phosphorus (NaH_2_PO_4_.2H_2_O, molar mass 156.01 g/mol, molar mass P 30.97 g/mol) | |
| --- | --- | --- | --- | --- |
|  | Total amount of N (mg) | Amount of compound in nutrient solution (mg/L) | Total amount of P (mg) | Amount of compound in nutrient solution (mg/L) |
| N limited | 13.5 | 2049.0 | 8.1 | 1020.15 |
| Balanced | 40.5 | 6147.3 | 2.7 | 340.05 |
| P limited | 121.5 | 18 441.96 | 0.9 | 113.35 |

|  | Total amount  (mg) | Molar masses of compounds  (g/mol) | Molar masses of elements  (g/mol) | Amount of compound in  nutrient solution (mg/L) |
| --- | --- | --- | --- | --- |
| K as KCl | 444 | 74.55 | 39.09 | 21 167.05 |
| Ca as CaCl_2_ . 2H_2_O | 110 | 147.01 | 40.08 | 10 088.04 |
| S and Mg as MgSO_4_.7H_2_O | 41 S / 63 Mg | 246.47 | 32.06 S / 24.31 Mg | 7 878.55 S / 15 973.63 Mg |
| Fe as FeSO_4_.7H_2_O | 10 | 278.01 | 55.85 | 1 244.40 |
| B as H_3_BO_3_ | 0.8 | 109.71 | 10.81 | 20.30 |
| Cu as CuSO_4_.5H_2_O | 0.06 | 249.68 | 63.55 | 5.89 |
| Mn as MnCl_2_.4H_2_O | 0.5 | 197.89 | 54.94 | 0.45 |
| Zn as ZnSO_4_.7H_2_O | 0.2 | 287.54 | 65.38 | 21.99 |
| Mo as Na_2_MoO_4_.2H_2_O | 0.1 | 241.96 | 95.95 | 6.30 |


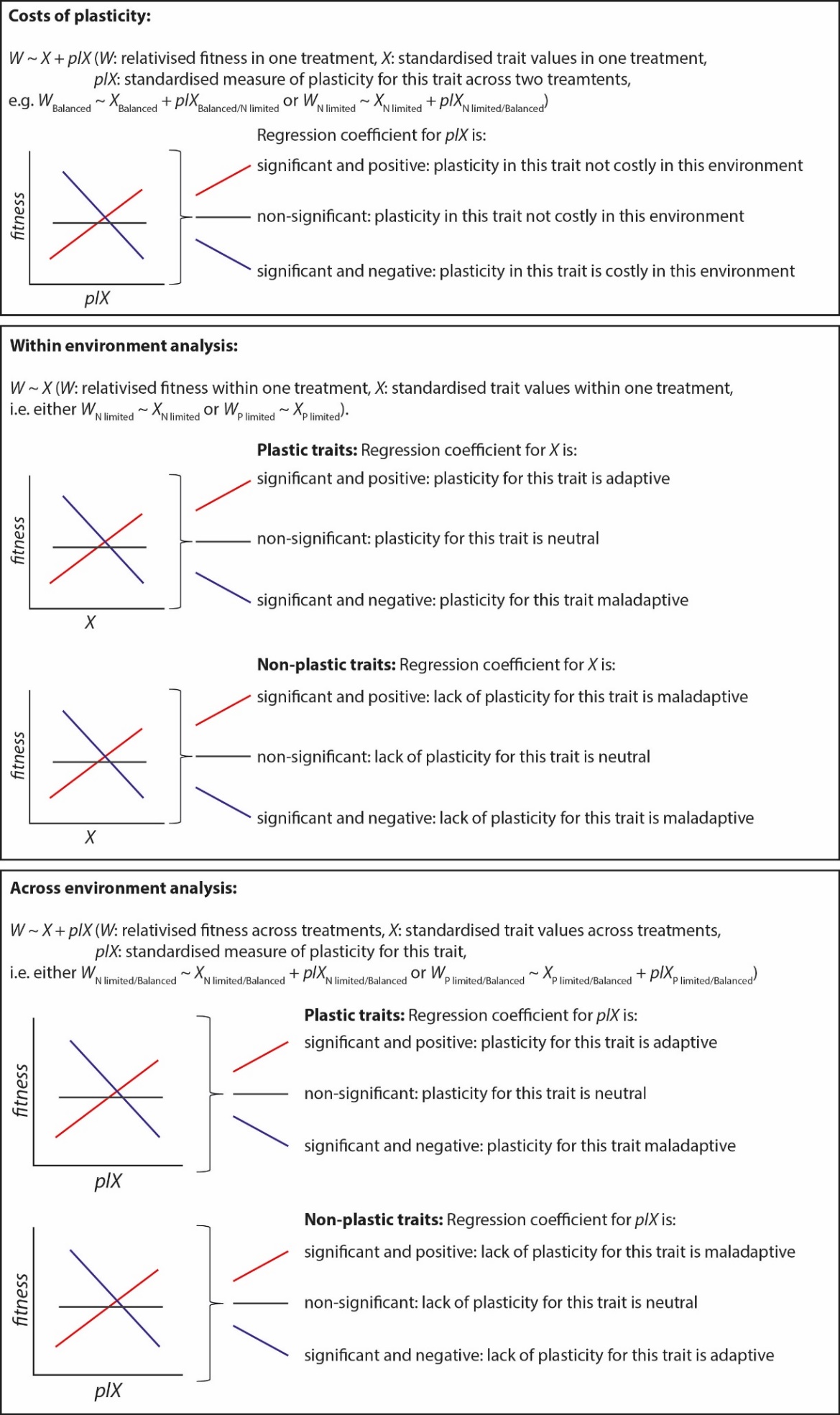


**Supplementary Fig. S1**: Conceptual explanation and interpretation of regression coefficients of the analyses for costs of plasticity (upper box), within environment analyses (middle box) and across environment analyses (lower box).


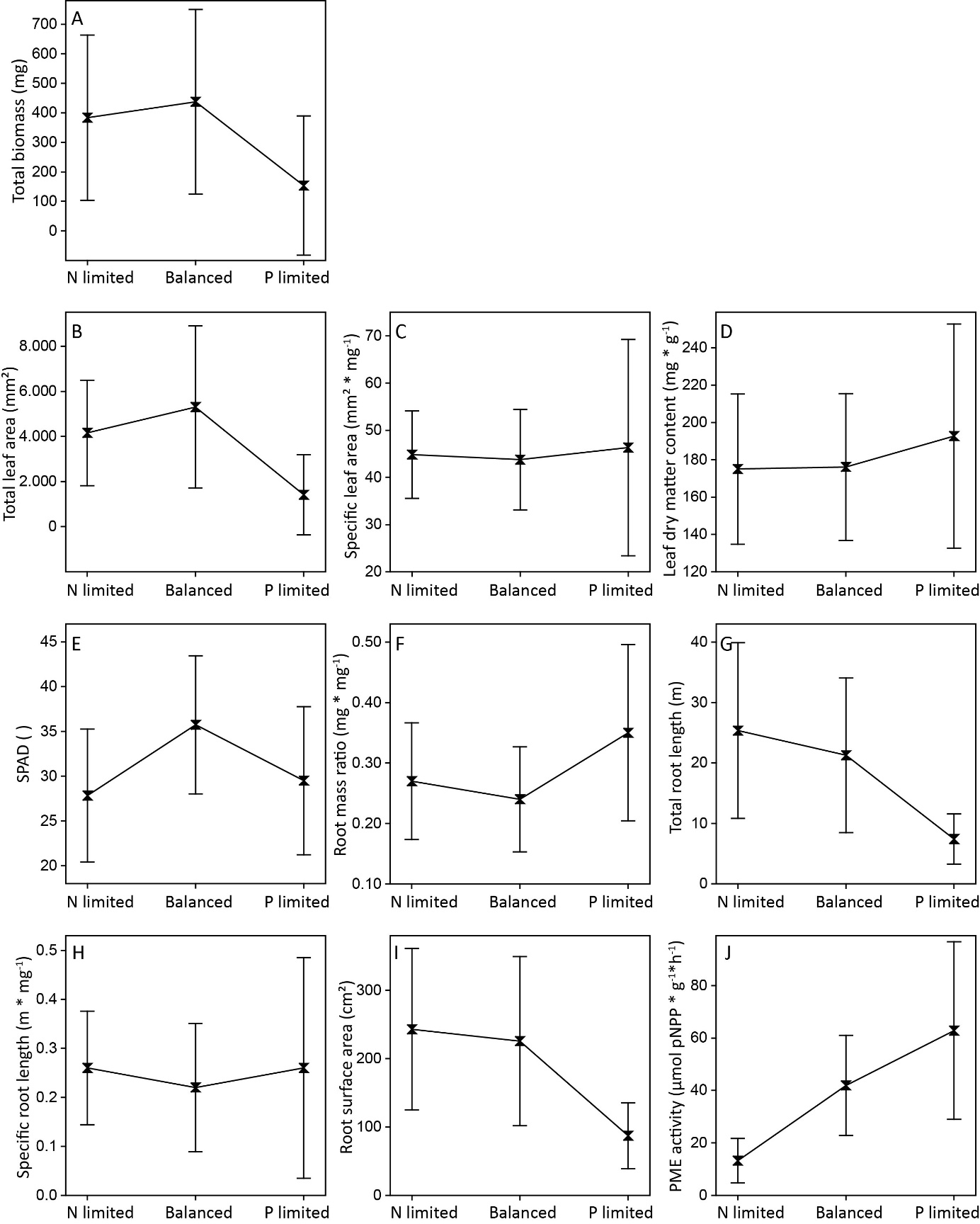


**Supplementary Fig. S2**: Mean response of fitness (Total biomass, A) and morphological and physiological traits to N limitation, balanced nutrient supply and P limitation. Given are means and standard deviations across all 17 species of this study. Leaf area (B), Specific leaf area (SLA, C), Leaf dry matter content (LDMC, D), SPAD (E), Root mass ratio (RMR, F), Total root length (G), Specific root length (SRL, H), Root surface area (I) and PME activity (J). For means and relative standard deviation (RSD) for each species separately see Supplementary Table S4, for each group of species Supplementary Table S5.

**Supplementary Table S3**: F-values and significance levels for one-way ANOVAs testing the effects of treatment (N limited vs balanced supply (upper part), or P limited vs balanced supply (lower part)) on fitness (total biomass) and other traits for each species separately. *p < 0.05. **p < 0.01 ***p < 0.001.

|  |  | N limitation versus Balanced nutrient supply | | | | | | | | | |
| --- | --- | --- | --- | --- | --- | --- | --- | --- | --- | --- | --- |
|  |  | Total biomass | Leaf area | SLA | LDMC | SPAD | RMR | Root length | SRL | Surface area | PME activity |
| Endangered | *E. fleischeri* | 2.18 | 3.21 | 0.001 | 0.41 | **20.26 **** | 1.43 | 0.32 | 1.51 | 0.47 | **25.03 **** |
|  | *B. secalinus* | 3.43 | 0.03 | 0.21 | 1.90 | 0.99 | 0.03 | 3.01 | 0.09 | 2.92 | **374.49 ***** |
|  | *B. squarrosus* | 1.34 | **8.68 **** | 0.24 | 1.65 | **10.11 **** | **5.79 *** | **16.11 ***** | **7.90 *** | **9.40 **** | **147.68 ***** |
|  | *L. remotum* | **12.61 **** | **32.35 ***** | 0.52 | 0.19 | **55.72 ***** | **22.09 ***** | **4.45 *** | **4.78 *** | 0.48 | **31.53 ***** |
|  | *L. temulentum* | **5.38 *** | **21.57 ***** | 2.17 | 0.03 | **72.01 ***** | **9.79 *** | 1.15 | 0.01 | 2.11 | **259.67 ***** |
|  | *T. subterraneum* | **10.21 **** | **4.92 *** | 0.12 | 1.68 | **36.290 ***** | 0.06 | 1.91 | 0.36 | 1.11 | **94.65 ***** |
|  |  |  |  |  |  |  |  |  |  |  |  |
| Non-endangered | *E. anagallidif.* | **5.32 *** | 2.73 | 0.05 | 2.97 | **13.59 *** | **20.33 ***** | 1.15 | **7.41 *** | 0.42 | **158.51 ***** |
|  | *B. hordeaceus* | **12.35 ***** | **17.63 ***** | 0.01 | 1.31 | **19.60 ***** | 1.53 | 0.07 | **4.49 *** | 1.54 | **87.01 ***** |
|  | *B. japonicus* | **17.17 ***** | **43.88 ***** | 0.99 | 1.19 | **57.84 ***** | **7.67 *** | 2.21 | 1.23 | 0.38 | **470.83 ***** |
|  | *H. murinum* | **12.61 **** | **32.34 ***** | 0.52 | 0.19 | **55.72 ***** | **22.09 ***** | **4.45 *** | **4.78 *** | 0.48 | **31.53 ***** |
|  | *M. lupulina* | 0.56 | 0.33 | 1.63 | 2.28 | 1.89 | 0.74 | 0.12 | 0.01 | 0.62 | **13.76 **** |
|  | *T. arvense* | 1.99 | 3.19 | 0.35 | 0.0002 | **16.20 **** | 0.001 | 0.62 | 1.63 | 0.01 | **112.01 ***** |
|  | *T. dubium* | **5.03 *** | 1.09 | 1.40 | 0.63 | **18.81 ***** | **7.29 *** | 1.89 | **9.48 **** | 1.51 | **216.47 ***** |
|  |  |  |  |  |  |  |  |  |  |  |  |
| Invasive | *A. sterilis* | 0.09 | 0.83 | 0.19 | 0.09 | **83.27 ***** | 2.30 | **10.59 **** | **6.68 *** | **11.77 **** | **274.04 ***** |
|  | *E. ciliatum* | 2.61 | 2.26 | 2.57 | 1.86 | **97.52 ***** | **16.59 ***** | 0.91 | **10.22 **** | **11.06 **** | **395.26 ***** |
|  | *H. jubatum* | **11.47 **** | **13.40 **** | 0.59 | 0.18 | **34.34 ***** | 3.39 | 0.78 | 0.05 | 1.17 | **66.22 ***** |
|  | *L. angustifolius* | 2.75 | 2.59 | 2.84 | **6.58 *** | **12.21 **** | 0.98 | 3.87 | 3.89 | 4.48 | **36.36 ***** |

|  | P limitation versus Balanced nutrient supply | | | | | | | | | | | |
| --- | --- | --- | --- | --- | --- | --- | --- | --- | --- | --- | --- | --- |
|  |  | Total biomass | Leaf area | SLA | LDMC | SPAD | RMR | Root length | SRL | Surface area | PME activity |  |
| Endangered | *E. fleischeri* | **37.79 ***** | **16.53 **** | 0.37 | 1.99 | **9.68 **** | **9.95 **** | 3.02 | 1.45 | 3.18 | 0.02 |  |
|  | *B. secalinus* | **51.61 ***** | **67.66 ***** | 3.48 | **4.87 *** | **46.47 ***** | 1.04 | **11.56 **** | 2.07 | **9.14 **** | **19.29 ***** |  |
|  | *B. squarrosus* | **82.39 ***** | **103.43 ***** | 0.14 | 0.64 | 1.32 | 3.26 | **26.04 ***** | **8.68 *** | **21.34 ***** | **45.39 ***** |  |
|  | *L. remotum* | **131.82 ***** | **296.76 ***** | 0.93 | **7.07 *** | **107.23 ***** | 4.12 | **69.14 ***** | 0.65 | **74.17 ***** | **19.06 ***** |  |
|  | *L. temulentum* | **121.79 ***** | **221.37 ***** | **23.25 **** | 1.99 | **63.89 ***** | **23.12 ***** | **115.09 ***** | **15.94 **** | **126.33 ***** | **13.08 **** |  |
|  | *T. subterraneum* | **109.06 ***** | **84.69 ***** | 1.00 | 0.06 | 0.79 | **16.24 ***** | **34.67 ***** | 1.29 | **35.17 ***** | **8.43 **** |  |
|  |  |  |  |  |  |  |  |  |  |  |  |  |
| Non-endangered | *E. anagallidif.* | **9.12 **** | **13.99 **** | **4.69 *** | **7.56 *** | 0.89 | **10.22 **** | **40.73 ***** | 2.37 | **66.38 ***** | 5.27 |  |
|  | *B. hordeaceus* | **117.43 ***** | **147.67 ***** | **7.12 ***** | **78.52 ***** | 0.07 | **14.98 **** | **16.24 **** | 0.89 | **20.95 ***** | **26.15 ***** |  |
|  | *B. japonicus* | **195.97 ***** | **217.81 ***** | 0.11 | 2.83 | **38.29 ***** | 0.22 | **129.95 ***** | 2.91 | **152.34 ***** | **21.14 ***** |  |
|  | *H. murinum* | **48.83 ***** | **46.53 ***** | **10.39 **** | 4.09 | 3.51 | **21.39 ***** | **15.84 ***** | 0.12 | **17.61 ***** | **73.48 ***** |  |
|  | *M. lupulina* | **5.23 *** | **7.09 *** | 0.23 | 1.44 | **10.66 **** | **6.22 *** | 5.13 | 0.34 | 4.68 | 0.36 |  |
|  | *T. arvense* | **34.36 ***** | **18.33 **** | 4.15 | 0.03 | **8.58 *** | **5.21 *** | **11.14 **** | 4.62 | **10.12 *** | **78.04 ***** |  |
|  | *T. dubium* | **86.78 ***** | **40.63 ***** | 0.03 | 0.30 | **18.53 ***** | **8.44 **** | **32.62 ***** | **15.58 **** | **33.98 ***** | **22.02 ***** |  |
|  |  |  |  |  |  |  |  |  |  |  |  |  |
| Invasive | *A. sterilis* | **172.35 ***** | **71.63 ***** | 0.34 | 1.50 | **22.59 ***** | **4.69 *** | **56.69 ***** | 1.19 | **43.29 ***** | **11.74 **** |  |
|  | *E. ciliatum* | **144.93 ***** | **149.83 ***** | **7.95 *** | **10.49 **** | **129.56 ***** | **66.74 ***** | **64.21 ***** | 1.85 | **105.82 ***** | **9.17 **** |  |
|  | *H. jubatum* | **156.02 ***** | **160.70 ***** | **17.16 ***** | **17.19 ***** | 4.12 | **6.46 *** | **47.81 ***** | 0.05 | **45.90 ***** | **57.28 ***** |  |
|  | *L. angustifolius* | 0.50 | **20.46 ***** | **6.69 *** | **19.86 ***** | 0.45 | 2.49 | 3.88 | **9.24 **** | 1.72 | 2.56 |  |

**Supplementary Table S4**: Mean and relative standard deviation (%) for each measured variable, calculated for each species and each group of replicates in each treatment (N limited, Balanced, P limited), respectively.

|  | **Total biomass** | **N limited** | **Balanced** | **P limited** |  |  | **Leaf area** | **N limited** | **Balanced** | **P limited** |
| --- | --- | --- | --- | --- | --- | --- | --- | --- | --- | --- |
| Endangered | Epi.fle | 28.38, 41.81 | 37.10, 31.71 | 11.35, 37.58 |  | Endangered | Epi.fle | 399.60, 80.11 | 705.95, 36.76 | 208.35, 45.14 |
|  | Bro.sec | 638.49, 15.85 | 531.96, 28.42 | 157.52, 41.62 |  |  | Bro.sec | 3618.40, 15.08 | 3562.41, 24.93 | 1093.97, 30.55 |
|  | Bro.squ | 263.76, 18.72 | 299.23, 27.89 | 51.83, 41.51 |  |  | Bro.squ | 4071.73, 18.44 | 5604.66, 26.11 | 812.41, 34.22 |
|  | Lol.rem | 490.37, 20.91 | 313.10, 19.08 | 84.72, 23.21 |  |  | Lol.rem | 2362.65, 18.69 | 2002.44, 14.44 | 249.61, 40.21 |
|  | Lol.tem | 619.16, 18.38 | 745.25, 17.28 | 235.48, 29.25 |  |  | Lol.tem | 3903.96, 18.32 | 5365.53, 12.89 | 1581.96, 25.91 |
|  | Tri.sub | 347.60, 14.75 | 449.99, 19.42 | 123.41, 28.69 |  |  | Tri.sub | 5191.52, 20.50 | 6658.99, 27.03 | 950.49, 50.14 |
|  |  |  |  |  |  |  |  |  |  |  |
| Non-endangered | Epi.ana | 104.48, 47.47 | 54.80, 85.18 | 10.06, 38.19 |  | Non-endangered | Epi.ana | 2163.06, 54.70 | 1321.43, 58.67 | 218.06, 21.41 |
|  | Bro.hor | 284.83, 21.79 | 413.93, 23.72 | 70.68, 28.03 |  |  | Bro.hor | 5202.31, 18.09 | 7946.74, 23.15 | 839.66, 22.41 |
|  | Bro.jap | 393.13, 19.38 | 544.98, 16.01 | 121.43, 32.28 |  |  | Bro.jap | 5799.72, 14.80 | 9785.79, 17.35 | 812.41, 34.22 |
|  | Hor.mur | 433.86, 21.40 | 686.93, 29.89 | 207.65, 33.60 |  |  | Hor.mur | 5902.77, 11.73 | 11427.90, 26.18 | 4164.59, 37.05 |
|  | Med.lup | 36.02, 99.80 | 50.98, 102.34 | 10.92, 44.91 |  |  | Med.lup | 820.43, 75.57 | 1019.80, 78.44 | 137.92, 56.30 |
|  | Tri.arv | 34.15, 37.23 | 44.96, 44.88 | 2.58, 114.34 |  |  | Tri.arv | 593.57, 33.47 | 854.61, 45.68 | 78.67, 138.91 |
|  | Tri.dub | 159.49, 26.61 | 215.75, 31.03 | 6.86, 58.28 |  |  | Tri.dub | 1908.07, 42.27 | 2333.81, 40.56 | 29.98, 70.41 |
|  |  |  |  |  |  |  |  |  |  |  |
| Invasive | Ave.ste | 927.5, 14.11 | 911.77, 11.67 | 356.14, 22.78 |  | Invasive | Ave.ste | 5433.49, 19.62 | 4957.14, 25.61 | 1371.27, 31.23 |
|  | Epi.cil | 441.23, 14.72 | 570.77, 17.86 | 111.95, 49.66 |  |  | Epi.cil | 6479.94, 9.85 | 6663.12, 36.42 | 898.43, 55.64 |
|  | Hor.jub | 374.44, 17.20 | 401.63, 39.04 | 48.67, 60.74 |  |  | Hor.jub | 4800.03, 11.73 | 6505.74, 20.92 | 841.94, 44.87 |
|  | Lup.ang | 928.04, 19.24 | 1077.64, 18.84 | 1005.46, 22.73 |  |  | Lup.ang | 8652.23, 31.80 | 10341.58, 14.73 | 6612.82, 29.45 |
|  |  |  |  |  |  |  |  |  |  |  |
|  | **SLA** | **N limited** | **Balanced** | **P limited** |  |  | **LDMC** | **N limited** | **Balanced** | **P limited** |
| Endangered | Epi.fle | 33.09, 38.52 | 33.35, 25.80 | 30.13, 34.82 |  | Endangered | Epi.fle | 112.26, 25.37 | 123.56, 23.51 | 152.40, 29.77 |
|  | Bro.sec | 44.72, 7.93 | 43.34, 20.44 | 50.46, 16.22 |  |  | Bro.sec | 208.61, 5.75 | 216.88, 6.77 | 198.48, 11.03 |
|  | Bro.squ | 42.75, 12.52 | 43.91, 12.04 | 43.05, 12.09 |  |  | Bro.squ | 173.37, 7.87 | 165.86, 7.53 | 170.30, 7.20 |
|  | Lol.rem | 39.08, 15.91 | 41.32, 11.73 | 37.29, 32.83 |  |  | Lol.rem | 227.83, 14.33 | 222.34, 6.73 | 262.10, 17.05 |
|  | Lol.tem | 43.57, 10.81 | 41.30, 3.09 | 48.04, 8.81 |  |  | Lol.tem | 195.59, 12.39 | 193.92, 7.39 | 204.33, 8.98 |
|  | Tri.sub | 42.73, 16.21 | 43.56, 7.74 | 40.22, 24.85 |  |  | Tri.sub | 167.79, 17.34 | 154.76, 8.29 | 152.37, 18.56 |
|  |  |  |  |  |  |  |  |  |  |  |
| Non-endangered | Epi.ana | 51.73, 11.73 | 52.53, 14.28 | 42.13, 26.07 |  | Non-endangered | Epi.ana | 135.30, 11.47 | 151.74, 13.55 | 229.60, 33.71 |
|  | Bro.hor | 47.35, 8.58 | 47.47, 4.68 | 43.26, 10.33 |  |  | Bro.hor | 124.43, 7.04 | 120.68, 4.58 | 160.22, 8.10 |
|  | Bro.jap | 41.27, 8.01 | 43.24, 12.33 | 44.06, 12.72 |  |  | Bro.jap | 170.59, 8.03 | 162.41, 11.90 | 178.73, 13.34 |
|  | Hor.mur | 41.34, 8.38 | 40.39, 5.71 | 47.87, 14.52 |  |  | Hor.mur | 178.33, 6.54 | 180.80, 7.42 | 165.09, 12.46 |
|  | Med.lup | 52.48, 35.16 | 42.09, 34.69 | 47.86, 62.73 |  |  | Med.lup | 167.97, 29.13 | 206.26, 27.04 | 258.15, 40.75 |
|  | Tri.arv | 48.76, 19.01 | 55.10, 56.17 | 117.95, 72.27 |  |  | Tri.arv | 155.43, 12.68 | 155.65, 24.85 | 166.90, 109.51 |
|  | Tri.dub | 58.89, 21.03 | 53.51, 13.00 | 54.71, 37.75 |  |  | Tri.dub | 192.98, 22.15 | 179.97, 15.56 | 187.57, 15.02 |
|  |  |  |  |  |  |  |  |  |  |  |
| Invasive | Ave.ste | 37.14, 8.22 | 38.10, 16.34 | 39.72, 15.47 |  | Invasive | Ave.ste | 208.45, 13.59 | 212.72, 15.99 | 229.13, 10.96 |
|  | Epi.cil | 50.04, 5.65 | 48.81, 8.56 | 42.18, 6.76 |  |  | Epi.cil | 214.24, 5.44 | 217.33, 9.01 | 259.01, 9.66 |
|  | Hor.jub | 43.87, 10.19 | 42.00, 13.64 | 54.96, 27.71 |  |  | Hor.jub | 176.38, 7.63 | 181.08, 11.79 | 149.91, 20.49 |
|  | Lup.ang | 40.50, 26.29 | 34.32, 7.90 | 30.84, 9.69 |  |  | Lup.ang | 108.80, 19.83 | 128.35, 5.87 | 148.07, 7.37 |
|  |  |  |  |  |  |  |  |  |  |  |
|  | **SPAD** | **N limited** | **Balanced** | **P limited** |  |  | **RMR** | **N limited** | **Balanced** | **P limited** |
| Endangered | Epi.fle | 21.66, 24.09 | 31.42, 7.94 | 23.55, 27.91 |  | Endangered | Epi.fle | 0.18, 20.70 | 0.14, 67.24 | 0.36, 47.36 |
|  | Bro.sec | 34.04, 5.59 | 34.84, 4.86 | 28.38, 8.72 |  |  | Bro.sec | 0.28, 27.32 | 0.29, 17.98 | 0.32, 29.15 |
|  | Bro.squ | 26.19, 11.13 | 30.33, 9.58 | 28.38, 14.55 |  |  | Bro.squ | 0.31, 13.28 | 0.25, 26.30 | 0.30, 21.97 |
|  | Lol.rem | 36.21, 5.80 | 41.05, 5.99 | 22.26, 17.10 |  |  | Lol.rem | 0.27, 34.38 | 0.20, 37.66 | 0.32, 49.34 |
|  | Lol.tem | 38.28, 4.71 | 44.47, 3.23 | 34.07, 11.31 |  |  | Lol.tem | 0.26, 14.80 | 0.20, 19.68 | 0.31, 18.09 |
|  | Tri.sub | 28.32, 6.87 | 39.12, 13.61 | 35.93, 27.40 |  |  | Tri.sub | 0.29, 15.54 | 0.30, 19.80 | 0.39, 9.39 |
|  |  |  |  |  |  |  |  |  |  |  |
| Non-endangered | Epi.ana | 22.81, 8.07 | 28.97, 13.98 | 26.96, 15.50 |  | Non-endangered | Epi.ana | 0.13, 19.70 | 0.25, 30.21 | 0.42, 34.19 |
|  | Bro.hor | 25.89, 6.86 | 29.42, 6.06 | 29.05, 13.64 |  |  | Bro.hor | 0.37, 17.92 | 0.33, 17.49 | 0.45, 16.36 |
|  | Bro.jap | 30.88, 6.12 | 38.30, 6.37 | 29.37, 13.14 |  |  | Bro.jap | 0.39, 14.28 | 0.30, 26.60 | 0.33, 43.75 |
|  | Hor.mur | 29.02, 9.99 | 36.41, 3.22 | 34.76, 7.30 |  |  | Hor.mur | 0.26, 5.31 | 0.17, 31.84 | 0.26, 10.59 |
|  | Med.lup | 34.04, 35.14 | 42.19, 18.71 | v27.03, 29.52 |  |  | Med.lup | 0.18, 31.34 | 0.16, 28.63 | 0.23, 25.94 |
|  | Tri.arv | 17.87, 16.61 | 30.43, 29.16 | 16.71, 44.05 |  |  | Tri.arv | 0.29, 43.35 | 0.28, 21.23 | 0.48, 51.46 |
|  | Tri.dub | 14.14, 33.83 | 23.86, 20.77 | 14.71, 21.13 |  |  | Tri.dub | 0.25, 25.58 | 0.19, 21.74 | 0.30, 37.88 |
|  |  |  |  |  |  |  |  |  |  |  |
| Invasive | Ave.ste | 31.63, 8.50 | 42.71, 6.40 | 35.60, 10.82 |  | Invasive | Ave.ste | 0.15, 8.99 | 0.13, 22.50 | 0.16, 14.80 |
|  | Epi.cil | 19.79, 11.43 | 27.89, 13.40 | 22.92, 29.56 |  |  | Epi.cil | 0.40, 19.55 | 0.34, 21.05 | 0.48, 32.58 |
|  | Hor.jub | 29.37, 10.31 | 40.05, 8.91 | 30.18, 6.55 |  |  | Hor.jub | 0.18, 13.68 | 0.27, 23.13 | 0.54, 17.13 |
|  | Lup.ang | 29.24, 32.40 | 45.21, 21.91 | 42.40, 17.99 |  |  | Lup.ang | 0.29, 20.18 | 0.26, 26.16 | 0.32, 24.75 |

**Supplementary Table S4** - *continued*

|  | **Root length** | **N limited** | **Balanced** | **P limited** |  |  | **SRL** | **N limited** | **Balanced** | **P limited** |
| --- | --- | --- | --- | --- | --- | --- | --- | --- | --- | --- |
| Endangered | Epi.fle | 1.33, 74.28 | 1.81, 59.19 | 0.73, 8.78 |  | Endangered | Epi.fle | 0.26, 28.24 | 0.58, 77.20 | 0.26, 19.95 |
|  | Bro.sec | 29.63, 24.70 | 22.02, 50.62 | 9.54, 33.85 |  |  | Bro.sec | 0.16, 32.80 | 0.15, 58.31 | 0.21, 50.70 |
|  | Bro.squ | 19.31, 18.34 | 12.99, 26.90 | 6.09, 35.49 |  |  | Bro.squ | 0.23, 17.46 | 0.18, 25.86 | 0.42, 60.01 |
|  | Lol.rem | 40.25, 18.23 | 17.27, 19.30 | 7.29, 19.36 |  |  | Lol.rem | 0.34, 37.59 | 0.30, 41.03 | 0.36, 43.72 |
|  | Lol.tem | 48.67, 15.64 | 44.51, 20.47 | 12.23, 22.25 |  |  | Lol.tem | 0.30, 19.01 | 0.30, 25.52 | 0.18, 33.11 |
|  | Tri.sub | 22.07, 27.16 | 26.92, 34.69 | 8.10, 26.96 |  |  | Tri.sub | 0.22, 16.37 | 0.20, 30.34 | 0.18, 27.09 |
|  |  |  |  |  |  |  |  |  |  |  |
| Non-endangered | Epi.ana | 36.89, 53.70 | 27.69, 24.93 | 1.34, 13.66 |  | Non-endangered | Epi.ana | 0.42, 22.80 | 0.30, 14.40 | 0.34, 4.14 |
|  | Bro.hor | 23.43, 20.52 | 22.49, 46.70 | 4.94, 14.14 |  |  | Bro.hor | 0.23, 28.18 | 0.16, 42.06 | 0.13, 23.38 |
|  | Bro.jap | 32.56, 21.12 | 28.55, 17.74 | 8.85, 23.14 |  |  | Bro.jap | 0.21, 19.48 | 0.18, 36.51 | 0.29, 63.57 |
|  | Hor.mur | 40.05, 37.01 | 27.76, 39.38 | 13.67, 17.52 |  |  | Hor.mur | 0.34, 24.40 | 0.25, 45.68 | 0.26, 26.17 |
|  | Med.lup | 4.77, 58.46 | 5.45, 61.10 | 0.95, 11.56 |  |  | Med.lup | 0.45, 17.44 | 0.44, 47.31 | 0.52, 31.95 |
|  | Tri.arv | 2.93, 62.14 | 3.61, 44.17 | 0.42, 23.32 |  |  | Tri.arv | 0.31, 38.65 | 0.25, 19.97 | 0.87, 102.4 |
|  | Tri.dub | 11.18, 27.74 | 9.24, 34.30 | 0.96, 40.72 |  |  | Tri.dub | 0.28, 19.08 | 0.22, 11.22 | 0.51, 46.11 |
|  |  |  |  |  |  |  |  |  |  |  |
| Invasive | Ave.ste | 30.18, 23.27 | 21.71, 19.72 | 10.74, 15.84 |  | Invasive | Ave.ste | 0.20, 17.06 | 0.17, 13.79 | 0.18, 18.21 |
|  | Epi.cil | 27.96, 15.52 | 31.34, 36.20 | 6.16, 31.76 |  |  | Epi.cil | 0.16, 35.70 | 0.16, 30.61 | 0.15, 69.98 |
|  | Hor.jub | 24.22, 26.15 | 25.63, 36.14 | 4.39, 52.43 |  |  | Hor.jub | 0.35, 22.87 | 0.25, 26.41 | 0.19, 43.72 |
|  | Lup.ang | 9.59, 27.89 | 7.28, 31.71 | 5.34, 34.39 |  |  | Lup.ang | 0.03, 35.40 | 0.02, 30.29 | 0.01, 26.21 |
|  |  |  |  |  |  |  |  |  |  |  |
|  | **Surface area** | **N limited** | **Balanced** | **P limited** |  |  | **PME activity** | **N limited** | **Balanced** | **P limited** |
| Endangered | Epi.fle | 15.18, 81.33 | 22.32, 58.97 | 8.75, 5.66 |  | Endangered | Epi.fle | 1.00, 71.68 | 9.33, 34.54 | 9.01, 27.81 |
|  | Bro.sec | 313.26, 23.97 | 235.65, 49.23 | 118.10, 34.41 |  |  | Bro.sec | 10.68, 24.85 | 60.36, 12.70 | 80.29, 15.10 |
|  | Bro.squ | 202.35, 23.55 | 140.53, 30.12 | 65.08, 39.69 |  |  | Bro.squ | 15.05, 9.71 | 45.78, 17.17 | 77.61, 16.36 |
|  | Lol.rem | 354.47, 17.34 | 153.28, 17.56 | 68.06, 19.14 |  |  | Lol.rem | 10.42, 24.95 | 27.29, 13.59 | 37.52, 17.09 |
|  | Lol.tem | 399.91, 16.71 | 449.20, 17.71 | 141.75, 23.94 |  |  | Lol.tem | 8.48, 28.77 | 31.02, 11.87 | 38.27, 13.47 |
|  | Tri.sub | 245.90, 28.58 | 284.87, 32.79 | 93.33, 28.05 |  |  | Tri.sub | 18.66, 16.49 | 52.20, 20.04 | 74.74, 29.44 |
|  |  |  |  |  |  |  |  |  |  |  |
| Non-endangered | Epi.ana | 343.03, 51.04 | 294.35, 19.37 | 16.65, 11.04 |  | Non-endangered | Epi.ana | 5.47, 28.98 | 29.36, 16.17 | 46.04, 38.47 |
|  | Bro.hor | 197.08, 20.22 | 237.62, 40.07 | 56.58, 17.78 |  |  | Bro.hor | 23.70, 21.64 | 59.64, 18.52 | 94.25, 18.01 |
|  | Bro.jap | 250.67, 19.23 | 263.52, 16.88 | 76.59, 23.15 |  |  | Bro.jap | 10.30, 15.97 | 43.69, 10.47 | 63.10, 19.86 |
|  | Hor.mur | 320.76, 35.59 | 286.98, 36.30 | 143.59, 19.97 |  |  | Hor.mur | 9.22, 17.85 | 18.26, 26.36 | 45.26, 19.25 |
|  | Med.lup | 51.01, 46.56 | 69.18, 65.99 | 10.25, 23.21 |  |  | Med.lup | 17.59, 17.87 | 52.46, 39.11 | 60.16, 22.80 |
|  | Tri.arv | 41.37, 66.54 | 40.40, 47.56 | 3.88, 20.48 |  |  | Tri.arv | 32.66, 21.17 | 83.02, 14.46 | 161.47, 9.38 |
|  | Tri.dub | 122.60, 23.45 | 105.53, 31.53 | 16.77, 26.06 |  |  | Tri.dub | 15.60, 15.84 | 55.17, 14.74 | 137.18, 41.81 |
|  |  |  |  |  |  |  |  |  |  |  |
| Invasive | Ave.ste | 327.67, 22.29 | 231.82, 21.44 | 120.51, 16.40 |  | Invasive | Ave.ste | 5.20, 20.75 | 25.04, 14.50 | 32.20, 17.14 |
|  | Epi.cil | 281.64, 11.60 | 321.12, 34.40 | 80.80, 23.99 |  |  | Epi.cil | 17.82, 23.10 | 50.79, 23.88 | 93.57, 14.02 |
|  | Hor.jub | 227.80, 22.06 | 291.13, 30.45 | 56.38, 48.48 |  |  | Hor.jub | 0.35, 86.18 | 21.85, 22.07 | 28.76, 24.90 |
|  | Lup.ang | 199.77, 21.68 | 155.41, 29.34 | 129.47, 29.35 |  |  | Lup.ang | 17.14, 45.08 | 39.35, 20.07 | 49.89, 36.32 |

Total biomass (mg), Leaf area (mm²), Specific leaf area (SRL, mm² * mg^-1^), Leaf dry matter content (LDMC, mg * g^-1^), SPAD ( ), Root mass ratio (RMR, mg * mg^-1^), Total root length (m), Specific root length (SRL, m * mg^-1^), Root surface area (cm²), Root phosphomonoesterase activity (PME activity, µmol pNPP * g^-1^ * h^-1^).

Endangered species: *Epilobium fleischeri* (Epi.fle)*, Bromus secalinus* (Bro.sec)*, Bromus squarrosus* (Bro.squ)*, Lolium remotum* (Lol.rem)*, Lolium temulentum* (Lol.tem)*, Trifolium subterraneum* (Tri.sub).

Non-endangered species: *Epilobium anagallidifolium* (Epi.ana)*, Bromus hordeaceus* (Bro.hor)*, Bromus japonicus* (Bro.jap)*, Hordeum murinum* (Hor.mur)*, Medicago lupulina* (Med.lup)*, Trifolium arvense* (Tri.arv)*, Trifolium dubium* (Tri.dub).

Invasive species: *Avena sterilis* (Ave.ste)*, Epilobium ciliatum* (Epi.cil)*, Hordeum jubatum* (Hor.jub)*, Lupinus angustifolius* (Lup.ang).

**Supplementary Table S5**: Mean and relative standard deviation (%) for each measured variable and for each treatment, calculated for all species combined and for each group of endangered, non-endangered and invasive species (for species names in each group, see Supplementary Table S4).

|  | **All species** | | |  |
| --- | --- | --- | --- | --- |
|  | **N limited** | **Balanced** | **P limited** |  |
| Total biomass | 383.63, 73.03 | 437.55, 71.42 | 153.40, 153.86 |  |
| Leaf area | 4153.98, 56.30 | 5303.13, 67.79 | 1411.32, 126.28 |  |
| SLA | 44.84, 20.68 | 43.76, 24.32 | 46.32, 49.47 |  |
| LDMC | 175.03, 23.02 | 176.13, 22.33 | 192.68, 31.16 |  |
| SPAD | 27.83, 26.63 | 35.73, 21.57 | 29.50, 28.02 |  |
| RMR | 0.27, 35.77 | 0.24, 35.57 | 0.35, 41.19 |  |
| Root length | 25.35, 57.34 | 21.27, 60.12 | 7.40, 56.28 |  |
| SRL | 0.26, 44.43 | 0.22, 58.52 | 0.26, 86.66 |  |
| Surface area | 243.06, 48.55 | 225.59, 54.87 | 87.18, 55.13 |  |
| PME activity | 13.21, 64.09 | 41.88, 45.57 | 62.81, 53.88 |  |
|  |  |  |  |  |
|  | **Endangered** | | |  |
|  | **N limited** | **Balanced** | **P limited** |  |
| Total biomass | 410.71, 53.89 | 408.49, 57.43 | 112.22, 75.43 |  |
| Leaf area | 3575.58, 40.17 | 4096.35, 56.40 | 881.63, 63.81 |  |
| SLA | 41.87, 15.91 | 41.40, 15.91 | 42.71, 23.96 |  |
| LDMC | 188.54, 20.32 | 181.49, 20.87 | 192.89, 24.07 |  |
| SPAD | 31.80, 18.36 | 37.06, 15.90 | 30.25, 23.53 |  |
| RMR | 0.27, 25.51 | 0.24, 34.97 | 0.34, 32.35 |  |
| Root length | 29.91, 47.86 | 23.45, 60.07 | 8.27, 43.97 |  |
| SRL | 0.26, 36.15 | 0.25, 62.96 | 0.27, 61.62 |  |
| Surface area | 284.14, 40.32 | 239.67, 59.40 | 93.26, 48.78 |  |
| PME activity | 11.80, 44.59 | 40.24, 42.03 | 58.40, 44.14 |  |
|  |  |  |  |  |
|  | **Non-endangered** | | | |
|  | **N limited** | **Balanced** | **P limited** |  |
| Total biomass | 206.57, 79.40 | 290.99, 90.55 | 64.83, 123.30 |  |
| Leaf area | 3345.13, 70.46 | 5251.76, 87.15 | 1265.33, 128.89 |  |
| SLA | 48.48, 22.43 | 47.68, 29.11 | 52.85, 64.03 |  |
| LDMC | 161.38, 21.20 | 164.67, 22.68 | 188.07, 39.48 |  |
| SPAD | 24.44, 30.46 | 32.51, 23.12 | 26.71, 29.31 |  |
| RMR | 0.27, 39.22 | 0.24, 35.42 | 0.35, 43.15 |  |
| Root length | 22.73, 73.00 | 18.69, 65.53 | 6.69, 79.70 |  |
| SRL | 0.30, 35.06 | 0.24, 45.83 | 0.35, 86.19 |  |
| Surface area | 195.85, 66.58 | 192.74, 60.67 | 67.94, 79.18 |  |
| PME activity | 16.53, 57.51 | 48.34, 44.46 | 77.38, 54.30 |  |
|  |  | | | |
|  | **Invasive** | | | |
|  | **N limited** | **Balanced** | **P limited** |  |
| Total biomass | 6282.18, 32.40 | 7183.47, 32.99 | 2361.41, 110.10 |  |
| Leaf area | 547.84, 69.21 | 579.38, 66.45 | 302.22, 51.92 |  |
| SLA | 42.95, 17.49 | 40.74, 16.67 | 41.87, 27.82 |  |
| LDMC | 178.71, 25.56 | 187.27, 21.72 | 199.03, 27.39 |  |
| SPAD | 27.46, 24.63 | 39.02, 21.88 | 32.59, 27.56 |  |
| RMR | 0.26, 43.04 | 0.25, 37.05 | 0.37, 46.85 |  |
| Root length | 23.33, 40.66 | 22.20, 50.76 | 6.95, 44.54 |  |
| SRL | 0.19, 63.19 | 0.15, 57.39 | 0.13, 72.72 |  |
| Surface area | 260.74, 27.03 | 256.14, 36.94 | 100.47, 37.89 |  |
| PME activity | 9.95, 87.36 | 33.91, 41.71 | 53.00, 55.00 |  |

Total biomass (mg), Leaf area (mm²), Specific leaf area (SRL, mm² * mg^-1^), Leaf dry matter content (LDMC, mg * g^-1^), SPAD ( ), Root mass ratio (RMR, mg * mg^-1^), Total root length (m), Specific root length (SRL, m * mg^-1^), Root surface area (cm²), Root phosphomonoesterase activity (PME activity, µmol pNPP * g^-1^ * h^-1^).

**Supplementary Table S6**: Analysis of costs of plasticity in morphological and physiological traits for each species separately. Costs were determined by regressing the mean fitness within one treatment (N limited, Balanced or P limited) on the mean of a trait in this treatment and a measure of plasticity across two treatments (N limited/balanced (left side) or P limited/balanced (right side), respectively). Given are regression coefficients for the trait values (*X*) in the specific environment. A significant negative regression coefficient for the plasticity term (*plX*) indicates that it is costly (positive and significant or non-significant: non-costly). An empty field indicates a non-plastic trait. *P<0.05, **P<0.01.

|  | **Endangered - *Epilobium fleischeri*** | | | | | | | |
| --- | --- | --- | --- | --- | --- | --- | --- | --- |
|  | Balanced | | N limited | | Balanced | | P limited | |
|  | *X (Bal)* | *plX (Bal-Nlim)* | *X (N lim)* | *plX (N lim-Bal)* | *X (Bal)* | *plX (Bal-P lim)* | *X (N lim)* | *plX (P lim-Bal)* |
| Leaf area |  |  |  |  | -0.070 | 0.386 | 0.324 | 0.078 |
| SLA |  |  |  |  |  |  |  |  |
| LDMC |  |  |  |  |  |  |  |  |
| SPAD | -0.617 | -0.126 | 0.158 | 0.022 | -0.181 | -0.101 | 0.301 | 0.514 |
| RMR |  |  |  |  | -0.281 | 0.233 | -0.201 | -0.034 |
| Root length |  |  |  |  |  |  |  |  |
| SRL |  |  |  |  |  |  |  |  |
| Surface area |  |  |  |  |  |  |  |  |
| PME activity | -0.400 | -0.030 | -0.245 | 0.100 |  |  |  |  |
|  |  |  |  |  |  |  |  |  |
|  | **Endangered – *Bromus secalinus*** | | | | | | | |
|  | Balanced | | N limited | | Balanced | | P limited | |
|  | *X (Bal)* | *plX (Bal-Nlim)* | *X (N lim)* | *plX (N lim-Bal)* | *X (Bal)* | *plX (Bal-P lim)* | *X (N lim)* | *plX (P lim-Bal)* |
| Leaf area |  |  |  |  | -0.112 | -0.008 | 0.257 | 0.002 |
| SLA |  |  |  |  |  |  |  |  |
| LDMC |  |  |  |  | 0.116 | -0.049 | 0.001 | -0.121 |
| SPAD |  |  |  |  | 0.141 | 0.087 | **0.449 **** | 0.161 |
| RMR |  |  |  |  |  |  |  |  |
| Root length |  |  |  |  | -0.074 | -0.011 | **0.353 **** | **-0.123 *** |
| SRL |  |  |  |  |  |  |  |  |
| Surface area |  |  |  |  | 0.038 | -0.023 | **0.362**** | 0.023 |
| PME activity | 0.136 | **-0.225 *** | -0.112 | 0.019 | 0.199 | 0.064 | -0.337 | -0.167 |
|  |  |  |  |  |  |  |  |  |
|  | **Endangered – *Bromus squarrosus*** | | | | | | | |
|  | Balanced | | N limited | | Balanced | | P limited | |
|  | *X (Bal)* | *plX (Bal-N lim)* | *X (N lim)* | *plX (N lim-Bal)* | *X (Bal)* | *plX (Bal-P lim)* | *X (P lim)* | *plX (P lim-Bal)* |
| Leaf area | **0.214 **** | -0.08 | 0.093 | 0.034 | **0.220 *** | -0.024 | 0.239 | 0.083 |
| SLA |  |  |  |  |  |  |  |  |
| LDMC |  |  |  |  |  |  |  |  |
| SPAD | -0.026 | **0.207 *** | -0.053 | 0.013 |  |  |  |  |
| RMR | -0.014 | -0.001 | 0.060 | 0.057 |  |  |  |  |
| Root length | **0.228 **** | 0.016 | **0.182 **** | -0.033 | **0.237 **** | -0.047 | **0.692 *** | -0.094 |
| SRL | 0.010 | -0.149 | -0.097 | -0.003 | -0.037 | -0.103 | **-0.464 *** | 0.079 |
| Surface area |  |  |  |  | **0.239 **** | -0.036 | -0.071 | -0.029 |
| PME activity | 0.072 | 0.076 | -0.111 | 0.078 | 0.074 | -0.062 | -0.213 | -0.117 |
|  |  |  |  |  |  |  |  |  |
|  | **Endangered – *Lolium remotum*** | | | | | | | |
|  | Balanced | | N limited | | Balanced | | P limited | |
|  | *X (Bal)* | *plX (Bal-N lim)* | *X (P lim)* | *plX (N lim-Bal)* | *X (Bal)* | *plX (Bal-P lim)* | *X (P lim)* | *plX (P lim-Bal)* |
| Leaf area | 0.054 | -0.088 | **0.145 *** | 0.115 | 0.058 | 0.116 | 0.092 | 0.078 |
| SLA |  |  |  |  |  |  |  |  |
| LDMC |  |  |  |  | 0.082 | 0.008 | -0.091 | -0.054 |
| SPAD | 0.046 | 0.02 | 0.066 | 0.054 | 0.203 | -0.015 | -0.055 | -0.001 |
| RMR | 0.083 | -0.05 | 0.013 | 0.139 |  |  |  |  |
| Root length | 0.113 | 0.025 | **0.151 *** | -0.039 | 0.108 | -0.101 | **0.194 *** | 0.024 |
| SRL | -0.102 | -0.001 | -0.036 | -0.003 |  |  |  |  |
| Surface area |  |  |  |  | 0.095 | -0.006 | 0.104 | 0.044 |
| PME activity | -0.047 | -0.005 | -0.012 | 0.121 | -0.028 | 0.09 | -0.067 | 0.036 |
|  |  |  |  |  |  |  |  |  |
|  | **Endangered – *Lolium temulentum*** | | | | | | | |
|  | Balanced | | N limited | | Balanced | | P limited | |
|  | *X (Bal)* | *plX (Bal-N lim)* | *X (P lim)* | *plX (N lim-Bal)* | *X (Bal)* | *plX (Bal-P lim)* | *X (P lim)* | *plX (P lim-Bal)* |
| Leaf area | **0.120 *** | 0.047 | 0.095 | -0.001 | **0.138 *** | 0.048 | **0.219 *** | -0.001 |
| SLA |  |  |  |  | -0.116 | -0.053 | **-0.155 *** | **-0.194 **** |
| LDMC |  |  |  |  |  |  |  |  |
| SPAD | 0.029 | -0.05 | 0.108 | -0.057 | 0.037 | -0.004 | 0.15 | -0.069 |
| RMR | 0.023 | **-0.130 **** | -0.012 | 0.017 | 0.083 | -0.074 | -0.115 | -0.05 |
| Root length |  |  |  |  | 0.047 | 0.039 | 0.146 | 0.093 |
| SRL |  |  |  |  | -0.069 | -0.010 | -0.146 | 0.057 |
| Surface area |  |  |  |  | 0.041 | -0.043 | 0.154 | 0.132 |
| PME activity | -0.058 | -0.034 | -0.099 | 0.072 | -0.058 | -0.071 | 0.164 | 0.100 |
|  |  |  |  |  |  |  |  |  |
|  | **Endangered – *Trifolium subterraneum*** | | | | | | | |
|  | Balanced | | N limited | | Balanced | | P limited | |
|  | *X (Bal)* | *plX (Bal-N lim)* | *X (P lim)* | *plX (N lim-Bal)* | *X (Bal)* | *plX (Bal-P lim)* | *X (P lim)* | *plX (P lim-Bal)* |
| Leaf area | **0.172 **** | -0.039 | 0.093 | 0.016 | **0.186 **** | -0.008 | **0.248 **** | -0.036 |
| SLA |  |  |  |  |  |  |  |  |
| LDMC |  |  |  |  |  |  |  |  |
| SPAD | -0.001 | -0.065 | 0.102 | 0.065 |  |  |  |  |
| RMR |  |  |  |  | -0.078 | 0.069 | **0.179 *** | **0.188 *** |
| Root length |  |  |  |  | **0.151 *** | -0.025 | 0.209 | 0.053 |
| SRL |  |  |  |  |  |  |  |  |
| Surface area |  |  |  |  | 0.061 | -0.001 | **0.200 *** | -0.127 |
| PME activity | -0.076 | -0.032 | 0.033 | -0.084 | -0.063 | -0.035 | 0.052 | -0.011 |

**Supplementary Table S6** - *continued*

|  | **Common – *Epilobium anagallidifolium*** | | | | | | | |
| --- | --- | --- | --- | --- | --- | --- | --- | --- |
|  | Balanced | | N limited | | Balanced | | P limited | |
|  | *X (Bal)* | *plX (Bal-Nlim)* | *X (N lim)* | *plX (N lim-Bal)* | *X (Bal)* | *plX (Bal-P lim)* | *X (N lim)* | *plX (P lim-Bal)* |
| Leaf area |  |  |  |  | **0.769 *** | 0.126 | -0.028 | -0.154 |
| SLA |  |  |  |  | -0.264 | 0.671 | **-0.091 *** | 0.056 |
| LDMC |  |  |  |  | 9.148 | 4.359 | -0.036 | -0.112 |
| SPAD | 2.867 | 0.27 | 0.013 | 0.344 |  |  |  |  |
| RMR | **-0.497 *** | 0.316 | -0.059 | 0.023 | **-0.628 *** | -0.142 | 0.054 | **-0.338 **** |
| Root length |  |  |  |  | 0.168 | 0.33 | 0.155 | 0.058 |
| SRL | 0.823 | -0.564 | 0.362 | 0.299 |  |  |  |  |
| Surface area |  |  |  |  | 0.746 | 0.209 | 0.113 | 0.168 |
| PME activity |  |  |  |  |  |  |  |  |
|  |  |  |  |  |  |  |  |  |
|  | **Common – *Bromus hordeaceus*** | | | | | | | |
|  | Balanced | | N limited | | Balanced | | P limited | |
|  | *X (Bal)* | *plX (Bal-Nlim)* | *X (N lim)* | *plX (N lim-Bal)* | *X (Bal)* | *plX (Bal-P lim)* | *X (N lim)* | *plX (P lim-Bal)* |
| Leaf area | **0.240 **** | -0.047 | **0.169 *** | 0.023 | **0.256 **** | **-0.072 **** | **0.220 *** | 0.066 |
| SLA |  |  |  |  | -0.063 | 0.032 | 0.001 | 0.150 |
| LDMC |  |  |  |  | -0.001 | 0.083 | 0.128 | -0.06 |
| SPAD | -0.044 | 0.015 | -0.078 | -0.034 |  |  |  |  |
| RMR |  |  |  |  | 0.003 | 0.121 | 0.163 | 0.030 |
| Root length |  |  |  |  | -0.017 | -0.061 | 0.237 | 0.122 |
| SRL | 0.040 | -0.096 | -0.095 | -0.100 |  |  |  |  |
| Surface area |  |  |  |  | 0.485 | 0.084 | 0.157 | -0.066 |
| PME activity | 0.054 | 0.050 | 0.022 | 0.049 | 0.111 | -0.151 | **-0.164 *** | 0.011 |
|  |  |  |  |  |  |  |  |  |
|  | **Common – *Bromus japonicus*** | | | | | | | |
|  | Balanced | | N limited | | Balanced | | P limited | |
|  | *X (Bal)* | *plX (Bal-N lim)* | *X (P lim)* | *plX (N lim-Bal)* | *X (Bal)* | *plX (Bal-P lim)* | *X (P lim)* | *plX (P lim-Bal)* |
| Leaf area | 0.057 | 0.041 | **0.151 *** | 0.004 | 0.064 | 0.021 | 0.125 | -0.022 |
| SLA |  |  |  |  |  |  |  |  |
| LDMC |  |  |  |  |  |  |  |  |
| SPAD | -0.019 | -0.028 | -0.023 | 0.039 | -0.014 | -0.016 | 0.032 | 0.111 |
| RMR | 0.052 | -0.049 | 0.040 | 0.045 |  |  |  |  |
| Root length |  |  |  |  | 0.091 | -0.013 | **0.261 **** | 0.016 |
| SRL |  |  |  |  |  |  |  |  |
| Surface area |  |  |  |  | **0.130 *** | -0.079 | **0.262 *** | -0.046 |
| PME activity | 0.003 | 0.035 | -0.020 | 0.073 | 0.007 | 0.040 | -0.124 | -0.091 |
|  |  |  |  |  |  |  |  |  |
|  | **Common – *Hordeum murinum*** | | | | | | | |
|  | Balanced | | N limited | | Balanced | | P limited | |
|  | *X (Bal)* | *plX (Bal-N lim)* | *X (P lim)* | *plX (N lim-Bal)* | *X (Bal)* | *plX (Bal-P lim)* | *X (P lim)* | *plX (P lim-Bal)* |
| Leaf area | **0.229 *** | -0.028 | 0.073 | -0.046 | **0.229 *** | 0.014 | **0.307 **** | -0.044 |
| SLA |  |  |  |  | -0.13 | -0.095 | 0.087 | **-0.266 *** |
| LDMC |  |  |  |  |  |  |  |  |
| SPAD | 0.116 | -0.002 | 0.032 | 0.008 |  |  |  |  |
| RMR | 0.136 | -0.156 | 0.001 | 0.089 | 0.134 | -0.127 | -0.021 | **-0.253 *** |
| Root Length | 0.084 | -0.152 | **0.202 *** | 0.049 | 0.040 | 0.224 | **0.220 *** | 0.055 |
| SRL | -0.094 | 0.018 | 0.033 | -0.041 |  |  |  |  |
| Surface Area |  |  |  |  | **0.169 **** | **0.189 **** | **0.270 *** | 0.032 |
| PME activity | 0.115 | 0.151 | -0.022 | -0.036 | **0.145 *** | **-0.210 *** | -0.107 | -0.090 |
|  |  |  |  |  |  |  |  |  |
|  | **Common – *Medicago lupulina*** | | | | | | | |
|  | Balanced | | N limited | | Balanced | | P limited | |
|  | *X (Bal)* | *plX (Bal-N lim)* | *X (P lim)* | *plX (N lim-Bal)* | *X (Bal)* | *plX (Bal-P lim)* | *X (P lim)* | *plX (P lim-Bal)* |
| Leaf area |  |  |  |  | **0.913 **** | 0.044 | 0.267 | **-0.481 *** |
| SLA |  |  |  |  |  |  |  |  |
| LDMC |  |  |  |  |  |  |  |  |
| SPAD |  |  |  |  | -0.435 | 1.534 | 0.595 | 0.171 |
| RMR |  |  |  |  | 0.188 | 0.365 | **-0.339 *** | 0.112 |
| Root length |  |  |  |  |  |  |  |  |
| SRL |  |  |  |  |  |  |  |  |
| Surface area |  |  |  |  |  |  |  |  |
| PME activity | -0.074 | 0.235 | -1.197 | 0.413 |  |  |  |  |
|  |  |  |  |  |  |  |  |  |
|  | **Common – *Trifolium arvense*** | | | | | | | |
|  | Balanced | | N limited | | Balanced | | P limited | |
|  | *X (Bal)* | *plX (Bal-N lim)* | *X (P lim)* | *plX (N lim-Bal)* | *X (Bal)* | *plX (Bal-P lim)* | *X (P lim)* | *plX (P lim-Bal)* |
| Leaf area |  |  |  |  | 0.340 | 0.172 | **1.440 **** | -0.089 |
| SLA |  |  |  |  |  |  |  |  |
| LDMC |  |  |  |  |  |  |  |  |
| SPAD | 0.454 | -0.101 | -0.037 | 0.121 | 0.301 | -0.169 | 0.762 | -1.276 |
| RMR |  |  |  |  | 0.193 | -0.390 | 0.411 | 0.161 |
| Root length |  |  |  |  | 0.390 | 0.019 | 1.690 | -0.528 |
| SRL |  |  |  |  |  |  |  |  |
| Surface area |  |  |  |  | 0.395 | 0.117 | 0.705 | 0.312 |
| PME activity | 0.095 | 0.057 | -0.071 | -0.087 | 3.471 | -1.756 | 1.440 | -0.089 |

**Supplementary Table S6** - *continued*

|  | **Common – *Trifolium dubium*** | | | | | | | |
| --- | --- | --- | --- | --- | --- | --- | --- | --- |
|  | Balanced | | N limited | | Balanced | | P limited | |
|  | *X (Bal)* | *plX (Bal-N lim)* | *X (N lim)* | *plX (N lim-Bal)* | *X (Bal)* | *plX (Bal-P lim)* | *X (N lim)* | *plX (P lim-Bal)* |
| Leaf area |  |  |  |  | 0.167 | -0.092 | 0.045 | -0.083 |
| SLA |  |  |  |  |  |  |  |  |
| LDMC |  |  |  |  |  |  |  |  |
| SPAD | 0.112 | -0.021 | 0.010 | -0.08 | 0.123 | -0.223 | -0.012 | 0.016 |
| RMR | -0.114 | 0.165 | -0.135 | 0.058 | -0.043 | 0.088 | 0.145 | -0.054 |
| Root length |  |  |  |  | **0.375 *** | 0.056 | 0.144 | 0.203 |
| SRL | 0.187 | -0.160 | 0.032 | **-0.202 *** | 0.016 | 0.015 | -0.048 | 0.101 |
| Surface area |  |  |  |  | **0.479 *** | -0.206 | 0.757 | -0.159 |
| PME activity | 0.114 | 0.146 | -0.014 | 0.027 | -0.027 | 0.123 | -0.076 | 0.002 |
|  |  |  |  |  |  |  |  |  |
|  | **Invasive – *Avena sterilis*** | | | | | | | |
|  | Balanced | | N limited | | Balanced | | P limited | |
|  | *X (Bal)* | *plX (Bal-N lim)* | *X (N lim)* | *plX (N lim-Bal)* | *X (Bal)* | *plX (Bal-P lim)* | *X (P lim)* | *plX (P lim-Bal)* |
| Leaf area |  |  |  |  | 0.025 | -0.045 | 0.145 | -0.027 |
| SLA |  |  |  |  |  |  |  |  |
| LDMC |  |  |  |  |  |  |  |  |
| SPAD | 0.060 | -0.044 | 0.052 | 0.023 | 0.039 | -0.036 | **0.145 *** | 0.056 |
| RMR |  |  |  |  | -0.041 | 0.069 | -0.097 | -0.029 |
| Root length | 0.016 | -0.019 | 0.098 | 0.066 | 0.045 | -0.069 | **0.166 *** | 0.019 |
| SRL | -0.052 | -0.043 | -0.029 | -0.048 |  |  |  |  |
| Surface area | -0.008 | **0.100 **** | 0.065 | -0.013 | 0.031 | -0.044 | **0.180 *** | -0.010 |
| PME activity | -0.016 | 0.001 | 0.053 | -0.086 | -0.018 | -0.006 | -0.080 | 0.025 |
|  |  |  |  |  |  |  |  |  |
|  | **Invasive – *Epilobium ciliatum*** | | | | | | | |
|  | Balanced | | N limited | | Balanced | | P limited | |
|  | *X (Bal)* | *plX (Bal-N lim)* | *X (N lim)* | *plX (N lim-Bal)* | *X (Bal)* | *plX (Bal-P lim)* | *X (P lim)* | *plX (P lim-Bal)* |
| Leaf area |  |  |  |  | **0.179 **** | 0.070 | **0.444 *** | -0.082 |
| SLA |  |  |  |  | 0.031 | -0.061 | 0.310 | -0.018 |
| LDMC |  |  |  |  | **0.169 *** | 0.081 | -0.33 | -0.076 |
| SPAD | 0.009 | -0.059 | 0.009 | -0.014 | 0.017 | 0.028 | 0.214 | 0.160 |
| RMR | -0.044 | 0.056 | -0.038 | -0.030 | -0.080 | 0.023 | 0.267 | 0.295 |
| Root length |  |  |  |  | 0.135 | 0.037 | 0.671 | 0.150 |
| SRL | 0.018 | 0.001 | -0.044 | 0.001 |  |  |  |  |
| Surface area | **0.155 *** | 0.004 | 0.085 | -0.004 | 0.193 | -0.061 | 0.814 | -0.068 |
| PME activity | 0.005 | 0.040 | 0.008 | 0.082 | 0.008 | 0.005 | -0.304 | 0.022 |
|  |  |  |  |  |  |  |  |  |
|  | **Invasive – *Hordeum jubatum*** | | | | | | | |
|  | Balanced | | N limited | | Balanced | | P limited | |
|  | *X (Bal)* | *plX (Bal-N lim)* | *X (P lim)* | *plX (N lim-Bal)* | *X (Bal)* | *plX (Bal-P lim)* | *X (P lim)* | *plX (P lim-Bal)* |
| Leaf area | 0.116 | 0.015 | 0.104 | 0.017 | 0.117 | -0.018 | **0.373 *** | 0.015 |
| SLA |  |  |  |  | -0.053 | 0.016 | 0.070 | **0.345 *** |
| LDMC |  |  |  |  | 0.006 | 0.030 | 0.014 | 0.161 |
| SPAD | 0.095 | -0.001 | 0.006 | 0.015 |  |  |  |  |
| RMR |  |  |  |  | -0.113 | 0.098 | 0.264 | -0.105 |
| Root length |  |  |  |  | 0.115 | 0.036 | **0.404 **** | -0.019 |
| SRL |  |  |  |  |  |  |  |  |
| Surface area |  |  |  |  | 0.082 | 0.030 | **0.364 *** | 0.016 |
| PME activity | 0.090 | -0.033 | -0.072 | 0.027 | 0.060 | -0.025 | -0.232 | 0.109 |
|  |  |  |  |  |  |  |  |  |
|  | **Invasive – *Lupinus angustifolius*** | | | | | | | |
|  | Balanced | | N limited | | Balanced | | P limited | |
|  | *X (Bal)* | *plX (Bal-N lim)* | *X (N lim)* | *plX (N lim-Bal)* | *X (Bal)* | *plX (Bal-P lim)* | *X (P lim)* | *plX (P lim-Bal)* |
| Leaf area |  |  |  |  | 0.114 | 0.005 | 0.240 | -0.104 |
| SLA |  |  |  |  | -0.047 | -0.004 | -0.053 | 0.119 |
| LDMC | 0.047 | -0.008 | 0.004 | 0.001 | 0.018 | -0.012 | 0.008 | 0.096 |
| SPAD | 0.039 | -0.082 | 0.107 | -0.04 |  |  |  |  |
| RMR |  |  |  |  |  |  |  |  |
| Root length |  |  |  |  |  |  |  |  |
| SRL |  |  |  |  | -0.136 | 0.079 | -0.109 | 0.064 |
| Surface area |  |  |  |  |  |  |  |  |
| PME activity | 0.101 | 0.041 | 0.006 | 0.074 |  |  |  |  |

**Supplementary Table S7**: Regression coefficients for within environment analyses of plastic and non-plastic traits within the N limited or the P limited treatment. Analyses were run for each species separately. Fitness in one treatment was regressed on each trait in this environment. Significant regression coefficients for the trait values (*X*) in the specific environment are in bold. *P<0.05, **P<0.01

|  | Within environment: N limited trt | | | |  | Within environment: P limited trt | | | |
| --- | --- | --- | --- | --- | --- | --- | --- | --- | --- |
|  | Endangered | Endangered | Endangered | Endangered |  | Endangered | Endangered | Endangered | Endangered |
| ***Plastic traits*** | *E. fleischeri* | *B. secalinus* | *B. squarrosus* | *L. remotum* |  | *E. fleischeri* | *B. secalinus* | *B. squarrosus* | *L. remotum* |
| Leaf area |  |  | 0.107 | 0.120 |  | 0.223 | 0.257 | **0.285 *** | 0.064 |
| SLA |  |  |  |  |  |  |  |  |  |
| LDMC |  |  |  |  |  |  | 0.072 |  | -0.081 |
| SPAD | 0.017 |  | -0.058 | 0.075 |  | -0.170 | **0.333 **** |  | **-0.058 **** |
| RMR |  |  | 0.076 | 0.052 |  | **-0.273 *** |  |  |  |
| Root length |  |  | **0.163 **** | **0.159 *** |  |  | **0.382 **** | 0.025 | 0.129 |
| SRL |  |  | -0.099 | -0.037 |  |  |  | **-0.240 *** |  |
| Surface area |  |  |  |  |  |  | **0.359 **** | -0.021 | 0.119 |
| PME activity | 0.014 | **-0.104 *** | -0.106 | -0.078 |  |  | -0.223 | -0.215 | -0.066 |
|  | Within environment: N limited trt | | | |  | Within environment: P limited trt | | | |
| ***Non-plastic traits*** | Endangered | Endangered | Endangered | Endangered |  | Endangered | Endangered | Endangered | Endangered |
|  | *E. fleischeri* | *B. secalinus* | *B. squarrosus* | *L. remotum* |  | *E. fleischeri* | *B. secalinus* | *B. squarrosus* | *L. remotum* |
| Leaf area | 0.027 | **0.143 **** |  |  |  |  |  |  |  |
| SLA | 0.003 | -0.028 | -0.114 | -0.064 |  | 0.042 | -0.157 | **-0.290 *** | -0.103 |
| LDMC | 0.024 | 0.026 | **0.126 *** | -0.029 |  | -0.143 |  | 0.067 |  |
| SPAD |  | 0.087 |  |  |  |  |  | 0.198 |  |
| RMR | 0.201 | **0.103 *** |  |  |  |  | **0.365 **** | 0.120 | 0.116 |
| Root length | 0.745 | 0.090 |  |  |  | 0.167 |  |  |  |
| SRL | **-0.748 *** | -0.074 |  |  |  | 0.097 | **-0.338 **** |  | **-0.202 **** |
| Surface area | 0.749 | 0.095 | **0.145 **** | **0.155 *** |  | 0.121 |  |  |  |
| PME activity |  |  |  |  |  | 0.359 |  |  |  |

|  | Within environment: N limited trt | | | |  | Within environment: P limited trt | | | |
| --- | --- | --- | --- | --- | --- | --- | --- | --- | --- |
|  | Endangered | Endangered | Common | Common |  | Endangered | Endangered | Common | Common |
| ***Plastic traits*** | *L. temulentum* | *T. subterraneum* | *E. anagallidifolium* | *B. hordeaceus* |  | *L. temulentum* | *T. subterraneum* | *E. anagallidifolium* | *B. hordeaceus* |
| Leaf area | 0.096 | **0.095 *** |  | **0.177 **** |  | **0.220 *** | **0.263 **** | -0.031 | **0.186 *** |
| SLA |  |  |  |  |  | -0.171 |  | -0.127 | -0.060 |
| LDMC |  |  |  |  |  |  |  | 0.057 | 0.136 |
| SPAD | 0.105 | 0.063 | -0.160 | -0.078 |  | 0.162 |  |  |  |
| RMR | -0.015 |  | -0.055 |  |  | -0.115 | 0.176 | -0.013 | 0.156 |
| Root length |  |  |  |  |  | 0.175 | 0.188 | 0.098 | **0.212 **** |
| SRL |  |  | 0.034 | **-0.170 **** |  | -0.152 |  |  |  |
| Surface area |  |  |  |  |  | 0.166 | **0.211 *** | 0.096 | **0.192 *** |
| PME activity | **-0.128 *** | -0.009 | 0.080 | 0.039 |  | **0.191 *** | -0.001 |  | -0.124 |
|  | Within environment: N limited trt | | | |  | Within environment: P limited trt | | | |
| ***Non-plastic traits*** | Endangered | Endangered | Common | Common |  | Endangered | Endangered | Common | Common |
|  | *L. temulentum* | *T. subterraneum* | *E. anagallidifolium* | *B. hordeaceus* |  | *L. temulentum* | *T. subterraneum* | *E. anagallidifolium* | *B. hordeaceus* |
| Leaf area |  |  | **0.411 **** |  |  |  |  |  |  |
| SLA | 0.058 | -0.027 | 0.199 | -0.059 |  |  | -0.189 |  |  |
| LDMC | -0.069 | 0.041 | -0.145 | 0.130 |  | 0.139 | 0.191 |  |  |
| SPAD |  |  |  |  |  |  | 0.144 | -0.013 | 0.133 |
| RMR |  | -0.016 |  | **0.143 *** |  |  |  |  |  |
| Root length | **0.167 **** | **0.123 **** | 0.251 | **0.152 *** |  |  |  |  |  |
| SRL | -0.056 | 0.080 |  |  |  |  | -0.161 | 0.058 | -0.169 |
| Surface area | **0.158 **** | **0.119 **** | 0.222 | **0.180 **** |  |  |  | 0.096 |  |
| PME activity |  |  |  |  |  |  |  |  |  |

|  | Within environment: N limited trt | | | |  |  | Within environment: P limited trt | | | |  |
| --- | --- | --- | --- | --- | --- | --- | --- | --- | --- | --- | --- |
|  | Common | Common | Common | Common | Common |  | Common | Common | Common | Common | Common |
| ***Plastic traits*** | *B. japonicus* | *H. murinum* | *M. lupulina* | *T. arvense* | *T. dubium* |  | *B. japonicus* | *H. murinum* | *M. lupulina* | *T. arvense* | *T. dubium* |
| Leaf area | **0.154 **** | 0.101 |  |  |  |  | 0.131 | **0.303 **** | 0.085 | **1.324 **** | 0.033 |
| SLA |  |  |  |  |  |  |  | 0.029 |  |  |  |
| LDMC |  |  |  |  |  |  |  |  |  |  |  |
| SPAD | -0.012 | 0.030 |  | -0.089 | -0.004 |  | 0.036 |  | **0.458 *** | 0.677 | -0.018 |
| RMR | 0.035 | 0.033 |  |  | -0.114 |  |  | -0.048 | **-0.364 **** | 0.588 | 0.074 |
| Root length |  | **0.170 *** |  |  |  |  | **0.265 **** | **0.238 *** |  | **1.785 **** | **0.682 *** |
| SRL |  | 0.052 |  |  | 0.012 |  |  |  |  |  | -0.413 |
| Surface area |  |  |  |  |  |  | **0.243 *** | **0.283 **** |  | 1.779 | **0.648 *** |
| PME activity | -0.057 | -0.016 | -0.832 | -0.084 | -0.024 |  | -0.102 | -0.088 |  | 0.143 | -0.078 |
|  | Within environment: N limited trt | | | |  |  | Within environment: P limited trt | | | |  |
| ***Non-plastic traits*** | Common | Common | Common | Common | Common |  | Common | Common | Common | Common | Common |
|  | *B. japonicus* | *H. murinum* | *M. lupulina* | *T. arvense* | *T. dubium* |  | *B. japonicus* | *H. murinum* | *M. lupulina* | *T. arvense* | *T. dubium* |
| Leaf area |  |  | **0.984 **** | 0.076 | **0.192 *** |  |  |  |  |  |  |
| SLA | -0.103 | -0.112 | **-0.726 *** | -0.110 | -0.036 |  | -0.137 |  | -0.215 | 0.466 | -0.033 |
| LDMC | 0.109 | 0.069 | **0.826 **** | 0.114 | 0.012 |  | -0.013 | -0.056 | -0.012 | -0.374 | 0.009 |
| SPAD |  |  | 0.767 |  |  |  |  | -0.110 |  |  |  |
| RMR |  |  | 0.044 | 0.201 |  |  | 0.192 |  |  |  |  |
| Root length | **0.172 **** |  | **1.043 **** | 0.298 | **0.212 **** |  |  |  | -0.004 |  |  |
| SRL | -0.017 |  | **-0.981 *** | -0.167 |  |  | **-0.241 *** | **-0.255 *** | -0.213 | -1.390 |  |
| Surface area | **0.178 **** | **0.177 **** | **1.022 **** | 0.266 | **0.235 **** |  |  |  | -0.135 |  |  |
| PME activity |  |  |  |  |  |  |  |  | 0.002 |  |  |

**Supplementary Table S7** - *continued*

|  | Within environment: N limited trt | | | |  | Within environment: P limited trt | | | |
| --- | --- | --- | --- | --- | --- | --- | --- | --- | --- |
|  | Invasive | Invasive | Invasive | Invasive |  | Invasive | Invasive | Invasive | Invasive |
| ***Plastic traits*** | *A. sterilis* | *E. ciliatum* | *H. jubatum* | *L. angustifolius* |  | *E. ciliatum* | *A. sterilis* | *H. jubatum* | *L. angustifolius* |
| Leaf area |  |  | **0.096 *** |  |  | **0.515 **** | 0.135 | **0.378 *** | **0.159 *** |
| SLA |  |  |  |  |  | 0.308 |  | -0.005 | -0.089 |
| LDMC |  |  |  | 0.014 |  | -0.270 |  | -0.003 | -0.009 |
| SPAD | 0.063 | 0.012 | 0.006 | 0.107 |  | 0.235 | **0.156 *** |  |  |
| RMR |  | -0.037 |  |  |  | 0.309 | -0.107 | 0.265 |  |
| Root length | 0.072 |  |  |  |  | **0.651 **** | **0.160 *** | **0.411 **** |  |
| SRL | -0.028 | -0.045 |  |  |  |  |  |  | -0.089 |
| Surface area | 0.069 | 0.083 |  |  |  | **0.610 *** | **0.181 **** | **0.372 *** |  |
| PME activity | -0.012 | 0.026 | -0.067 | 0.018 |  | -0.317 | -0.069 | -0.251 |  |
|  | Within environment: N limited trt | | | |  | Within environment: P limited trt | | | |
|  | Invasive | Invasive | Invasive | Invasive |  | Invasive | Invasive | Invasive | Invasive |
| ***Non-plastic traits*** | *A. sterilis* | *E. ciliatum* | *H. jubatum* | *L. angustifolius* |  | *E. ciliatum* | *A. sterilis* | *H. jubatum* | *L. angustifolius* |
| Leaf area | 0.051 | 0.091 |  | 0.100 |  |  |  |  |  |
| SLA | -0.051 | **-0.111 *** | 0.023 | -0.008 |  |  | **-0.148 *** |  |  |
| LDMC | 0.025 | **0.136 **** | 0.020 |  |  |  | 0.080 |  |  |
| SPAD |  |  |  |  |  |  |  | **0.382 **** | 0.083 |
| RMR | 0.031 |  | 0.091 | **0.131 *** |  |  |  |  | 0.074 |
| Root length |  | 0.057 | 0.019 | 0.108 |  |  |  |  | **0.163 *** |
| SRL |  |  | **-0.109 *** | -0.120 |  | -0.474 | -0.080 | **-0.329 *** |  |
| Surface area |  |  | 0.076 | **0.144 *** |  |  |  |  | **0.188 **** |
| PME activity |  |  |  |  |  |  |  |  | -0.083 |

**Supplementary Table S8**: Across environment analyses of adaptive plasticity (for plastic traits) and whether a lack of plasticity is adaptive, maladaptive or neutral (for non-plastic traits). Analyses were run for each species separately. Fitness across two treatments (i.e. either N limited/Balanced or P limited/Balanced) was regressed on each trait (*X*) and a measure of plasticity (*plX*) across those treatments. Significant (P<0.05) coefficients are in bold. *P<0.05, **P<0.01

|  | Across environment analyses: N limited versus balanced treatment | | | | | | | |
| --- | --- | --- | --- | --- | --- | --- | --- | --- |
|  | Endangered | | Endangered | | Endangered | | Endangered | |
|  | *E. fleischeri* | | *B. secalinus* | | *B. squarrosus* | | *L. remotum* | |
| ***Plastic traits*** | *X (Nlim-Bal)* | *plX (Nlim-Bal)* | *X (Nlim-Bal)* | *plX (Nlim-Bal)* | *X (Nlim-Bal)* | *plX (Nlim-Bal)* | *X (Nlim-Bal)* | *plX (Nlim-Bal)* |
| Leaf area |  |  |  |  | **0.183 **** | -0.030 | 0.197 | 0.040 |
| SLA |  |  |  |  |  |  |  |  |
| LDMC |  |  |  |  |  |  |  |  |
| SPAD | 0.009 | -0.067 |  |  | 0.019 | **0.128 *** | -0.138 | 0.071 |
| RMR |  |  |  |  | -0.032 | 0.041 | 0.132 | 0.046 |
| Root length |  |  |  |  | 0.087 | 0.037 | 0.266 | -0.020 |
| SRL |  |  |  |  | -0.071 | -0.072 | -0.029 | 0.001 |
| Surface area |  |  |  |  |  |  |  |  |
| PME activity | 0.034 | -0.089 | -0.086 | -0.081 | 0.074 | 0.082 | -0.228 | 0.055 |
|  |  |  |  |  |  |  |  |  |
|  | Across environment analyses: N limited versus balanced treatment | | | | | | | |
|  | Endangered | | Endangered | | Endangered | | Endangered | |
|  | *E. fleischeri* | | *B. secalinus* | | *B. squarrosus* | | *L. remotum* | |
| ***Non-plastic traits*** | *X (Nlim-Bal)* | *plX (Nlim-Bal)* | *X (Nlim-Bal)* | *plX (Nlim-Bal)* | *X (Nlim-Bal)* | *plX (Nlim-Bal)* | *X (Nlim-Bal)* | *plX (Nlim-Bal)* |
| Leaf area | **0.202 *** | **0.210 *** | **0.167 **** | -0.008 |  |  |  |  |
| SLA | -0.117 | -0.016 | -0.054 | 0.072 | -0.069 | -0.022 | -0.145 | 0.036 |
| LDMC | -0.093 | 0.095 | -0.071 | 0.033 | 0.036 | -0.063 | 0.003 | -0.064 |
| SPAD |  |  | 0.089 | -0.039 |  |  |  |  |
| RMR | -0.071 | 0.044 | **0.123 *** | -0.007 |  |  |  |  |
| Root length | 0.260 | **-0.415 *** | 0.098 | 0.086 |  |  |  |  |
| SRL | 0.307 | 0.215 | -0.065 | -0.071 |  |  |  |  |
| Surface area | 0.460 | -0.088 | **0.145 **** | **-0.112 *** | **0.101 *** | **0.123 *** | 0.257 | **0.071 *** |
| PME activity |  |  |  |  |  |  |  |  |
|  |  |  |  |  |  |  |  |  |
|  | Across environment analyses: P limited versus balanced treatment | | | | | | | |
|  | Endangered | | Endangered | | Endangered | | Endangered | |
|  | *E. fleischeri* | | *B. secalinus* | | *B. squarrosus* | | *L. remotum* | |
| ***Plastic traits*** | *X (Plim-Bal)* | *plX (Plim-Bal)* | *X (Plim-Bal)* | *plX (Plim-Bal)* | *X (Plim-Bal)* | *plX (Plim-Bal)* | *X (Plim-Bal)* | *plX (Plim-Bal)* |
| Leaf area | **0.497 *** | 0.109 | **0.600 **** | -0.019 | **0.771 **** | -0.010 | 0.594 | 0.115 |
| SLA |  |  |  |  |  |  |  |  |
| LDMC |  |  | 0.217 | 0.007 |  |  | -0.329 | -0.032 |
| SPAD | 0.374 | 0.165 | **0.586 **** | 0.137 |  |  | 0.365 | -0.016 |
| RMR | **-0.584 **** | 0.184 |  |  |  |  |  |  |
| Root length |  |  | **0.437 **** | 0.015 | **0.724 **** | -0.040 | 0.557 | -0.102 |
| SRL |  |  |  |  | **-0.776 **** | -0.114 |  |  |
| Surface area |  |  | **0.429 **** | 0.005 | **0.684 **** | -0.050 | 0.595 | 0.010 |
| PME activity |  |  | **-0.455 **** | -0.083 | **-0.614 **** | -0.167 | -0.451 | 0.070 |
|  |  | | | | | | | |
|  | Across environment analyses: P limited versus balanced treatment | | | | | | | |
|  | Endangered | | Endangered | | Endangered | | Endangered | |
|  | *E. fleischeri* | | *B. secalinus* | | *B. squarrosus* | | *L. remotum* | |
| ***Non-plastic traits*** | *X (Plim-Bal)* | *plX (Plim-Bal)* | *X (Plim-Bal)* | *plX (Plim-Bal)* | *X (Plim-Bal)* | *plX (Plim-Bal)* | *X (Plim-Bal)* | *plX (Plim-Bal)* |
| Leaf area |  |  |  |  |  |  |  |  |
| SLA | -0.139 | 0.039 | **-0.337 *** | -0.009 | -0.037 | 0.052 | 0.098 | 0.067 |
| LDMC | **-0.432 *** | -0.291 |  |  | -0.128 | -0.027 |  |  |
| SPAD |  |  |  |  | 0.249 | 0.396 |  |  |
| RMR |  |  | 0.027 | -0.131 | -0.290 | -0.100 | -0.195 | -0.040 |
| Root length | 0.385 | 0.237 |  |  |  |  |  |  |
| SRL | 7.269 | -1.194 | **-0.362 *** | 0.066 |  |  | -0.298 | 0.060 |
| Surface area | 0.646 | 0.186 |  |  |  |  |  |  |
| PME activity | -0.142 | -0.007 |  |  |  |  |  |  |

**Supplementary Table S8** - *continued*

|  | Across environment analyses: N limited versus balanced treatment | | | | | | | | |
| --- | --- | --- | --- | --- | --- | --- | --- | --- | --- |
|  | Endangered | | Endangered | | Common | | Common | |  |
|  | *L. temulentum* | | *T. subterraneum* | | *E. anagallidifolium* | | *B. hordeaceus* | |  |
| ***Plastic traits*** | *X (Nlim-Bal)* | *plX (Nlim-Bal)* | *X (Nlim-Bal)* | *plX (Nlim-Bal)* | *X (Nlim-Bal)* | *plX (Nlim-Bal)* | *X (Nlim-Bal)* | *plX (Nlim-Bal)* |  |
| Leaf area | **0.146 **** | 0.028 | **0.193 **** | -0.015 |  |  | **0.284 **** | -0.014 |  |
| SLA |  |  |  |  |  |  |  |  |  |
| LDMC |  |  |  |  |  |  |  |  |  |
| SPAD | **0.115 **** | -0.050 | **0.106 *** | -0.046 | -0.165 | 0.224 | 0.100 | 0.026 |  |
| RMR | -0.051 | -0.068 |  |  | **-0.420 **** | 0.118 |  |  |  |
| Root length |  |  |  |  |  |  |  |  |  |
| SRL |  |  |  |  | 0.222 | -0.066 | -0.125 | -0.042 |  |
| Surface area |  |  |  |  |  |  |  |  |  |
| PME activity | 0.078 | 0.049 | **0.099 *** | -0.037 | -0.137 | -0.234 | **0.192 **** | 0.044 |  |
|  |  |  |  |  |  |  |  |  |  |
|  | Across environment analyses: N limited versus balanced treatment | | | | | | | | |
|  | Endangered | | Endangered | | Common | | Common | |  |
|  | *L. temulentum* | | *T. subterraneum* | | *E. anagallidifolium* | | *B. hordeaceus* | |  |
| ***Non-plastic traits*** | *X (Nlim-Bal)* | *plX (Nlim-Bal)* | *X (Nlim-Bal)* | *plX (Nlim-Bal)* | *X (Nlim-Bal)* | *plX (Nlim-Bal)* | *X (Nlim-Bal)* | *plX (Nlim-Bal)* |  |
| Leaf area |  |  |  |  | **0.581 **** | -0.027 |  |  |  |
| SLA | -0.038 | -0.042 | -0.017 | -0.071 | -0.041 | 0.006 | -0.038 | 0.053 |  |
| LDMC | -0.002 | -0.039 | -0.001 | 0.023 | -0.188 | -0.058 | -0.004 | 0.104 |  |
| SPAD |  |  |  |  |  |  |  |  |  |
| RMR |  |  | -0.082 | -0.065 |  |  | 0.061 | 0.075 |  |
| Root length | 0.045 | 0.044 | **0.174 **** | 0.024 | 0.280 | 0.272 | **0.162 *** | 0.021 |  |
| SRL | -0.069 | 0.042 | 0.059 | 0.024 |  |  |  |  |  |
| Surface area | **0.108 *** | 0.019 | **0.154 **** | 0.023 | 0.157 | -0.129 | **0.235 **** | 0.065 |  |
| PME activity |  |  |  |  |  |  |  |  |  |
|  |  |  |  |  |  |  |  |  |  |
|  | Across environment analyses: P limited versus balanced treatment | | | | | | | | |
|  | Endangered | | Endangered | | Common | | Common | |  |
|  | *L. temulentum* | | *T. subterraneum* | | *E. anagallidifolium* | | *B. hordeaceus* | |  |
| ***Plastic traits*** | *X (Plim-Bal)* | *plX (Plim-Bal)* | *X (Plim-Bal)* | *plX (Plim-Bal)* | *X (Plim-Bal)* | *plX (Plim-Bal)* | *X (Plim-Bal)* | *plX (Plim-Bal)* |  |
| Leaf area | **0.555 **** | 0.032 | **0.596 **** | -0.008 | **1.278 **** | 0.078 | **0.778 **** | -0.043 |  |
| SLA | **-0.457 **** | -0.066 |  |  | 0.370 | 0.370 | **0.377 *** | 0.128 |  |
| LDMC |  |  |  |  | -0.487 | 0.045 | **-0.644 **** | 0.042 |  |
| SPAD | **0.501 **** | 0.002 |  |  |  |  |  |  |  |
| RMR | **-0.410 **** | 0.035 | **-0.416 **** | 0.065 | **-0.694 *** | -0.111 | **-0.444 *** | 0.126 |  |
| Root length | **0.522 **** | 0.093 | **0.558 **** | -0.064 | **0.960 **** | 0.202 | **0.981 *** | 0.014 |  |
| SRL | **0.309 *** | -0.066 |  |  |  |  |  |  |  |
| Surface area | **0.522 **** | 0.001 | **0.575 **** | -0.065 | 0.988 | 0.311 | **1.028 **** | 0.071 |  |
| PME activity | **-0.329 *** | 0.008 | **-0.359 *** | 0.006 |  |  | **-0.647 *** | 0.210 |  |
|  |  | | | | | | | | |
|  | Across environment analyses: P limited versus balanced treatment | | | | | | | | |
|  | Endangered | | Endangered | | Common | | Common | |  |
|  | *L. temulentum* | | *T. subterraneum* | | *E. anagallidifolium* | | *B. hordeaceus* | |  |
| ***Non-plastic traits*** | *X (Plim-Bal)* | *plX (Plim-Bal)* | *X (Plim-Bal)* | *plX (Plim-Bal)* | *X (Plim-Bal)* | *plX (Plim-Bal)* | *X (Plim-Bal)* | *plX (Plim-Bal)* |  |
| Leaf area |  |  |  |  |  |  |  |  |  |
| SLA |  |  | 0.061 | 0.006 |  |  |  |  |  |
| LDMC | -0.096 | -0.020 | 0.157 | -0.059 |  |  |  |  |  |
| SPAD |  |  | 0.141 | 0.005 | -0.146 | -0.078 | 0.063 | -0.101 |  |
| RMR |  |  |  |  |  |  |  |  |  |
| Root length |  |  |  |  |  |  |  |  |  |
| SRL |  |  | 0.243 | -0.056 | -0.225 | -0.594 | 0.219 | 0.140 |  |
| Surface area |  |  |  |  |  |  |  |  |  |
| PME activity |  |  |  |  | -0.855 | 0.323 |  |  |  |

**Supplementary Table S8** - *continued*

|  | Across environment analyses: N limited versus balanced treatment | | | | | | | | | | |
| --- | --- | --- | --- | --- | --- | --- | --- | --- | --- | --- | --- |
|  | Common | | Common | | Common | | Common | | Common | |  |
| ***Plastic traits*** | *B. japonicus* | | *H. murinum* | | *M. lupulina* | | *T. arvense* | | *T. dubium* | |  |
|  | *X (Nlim-Bal)* | *plX (Nlim-Bal)* | *X (Nlim-Bal)* | *plX (Nlim-Bal)* | *X (Nlim-Bal)* | *plX (Nlim-Bal)* | *X (Nlim-Bal)* | *plX (Nlim-Bal)* | *X (Nlim-Bal)* | *plX (Nlim-Bal)* |  |
| Leaf area | **0.183 **** | 0.033 | **0.304 **** | -0.036 |  |  |  |  |  |  |  |
| SLA |  |  |  |  |  |  |  |  |  |  |  |
| LDMC |  |  |  |  |  |  |  |  |  |  |  |
| SPAD | **0.141 **** | 0.005 | **0.230 **** | 0.032 |  |  | 0.186 | 0.136 | 0.137 | -0.051 |  |
| RMR | -0.051 | -0.028 | -0.097 | -0.049 |  |  |  |  | **-0.193 *** | 0.132 |  |
| Root length |  |  | -0.074 | -0.202 |  |  |  |  |  |  |  |
| SRL |  |  | -0.167 | -0.047 |  |  |  |  | -0.037 | -0.114 |  |
| Surface area |  |  |  |  |  |  |  |  |  |  |  |
| PME activity | **0.162 **** | 0.050 | **0.248 **** | 0.081 | -0.469 | -0.074 | 0.186 | -0.026 | **0.175 *** | 0.105 |  |
|  |  |  |  |  |  |  |  |  |  |  |  |
|  | Across environment analyses: N limited versus balanced treatment | | | | | | | | | | |
| ***Non-plastic traits*** | Common | | Common | | Common | | Common | | Common | |  |
|  | *B. japonicus* | | *H. murinum* | | *M. lupulina* | | *T. arvense* | | *T. dubium* | |  |
|  | *X (Nlim-Bal)* | *plX (Nlim-Bal)* | *X (Nlim-Bal)* | *plX (Nlim-Bal)* | *X (Nlim-Bal)* | *plX (Nlim-Bal)* | *X (Nlim-Bal)* | *plX (Nlim-Bal)* | *X (Nlim-Bal)* | *plX (Nlim-Bal)* |  |
| Leaf area |  |  |  |  | **0.867 **** | -0.206 | **0.336 **** | 0.052 | **0.229 **** | 0.118 |  |
| SLA | -0.016 | 0.040 | -0.162 | -0.039 | **-0.813 *** | 0.268 | -0.201 | 0.027 | -0.070 | -0.114 |  |
| LDMC | 0.044 | -0.006 | 0.068 | -0.097 | **0.784 **** | 0.295 | 0.057 | -0.141 | -0.035 | 0.108 |  |
| SPAD |  |  |  |  | -0.126 | 0.473 |  |  |  |  |  |
| RMR |  |  |  |  | 0.070 | 0.169 | 0.211 | 0.125 |  |  |  |
| Root length | 0.062 | -0.018 | -0.074 | -0.202 | **0.926 *** | -0.315 | **0.441 **** | 0.009 | **0.180 *** | 0.007 |  |
| SRL | -0.093 | 0.017 | -0.167 | -0.047 | **-0.920 **** | -0.382 | -0.184 | 0.077 |  |  |  |
| Surface area | **0.145 **** | -0.025 | 0.160 | -0.063 | 0.086 | -0.797 | 0.272 | 0.061 | **0.171 *** | -0.061 |  |
| PME activity |  |  |  |  |  |  |  |  |  |  |  |
|  |  |  |  |  |  |  |  |  |  |  |  |
|  | Across environment analyses: P limited versus balanced treatment | | | | | | | | | | |
| ***Plastic traits*** | Common | | Common | | Common | | Common | | Common | |  |
|  | *B. japonicus* | | *H. murinum* | | *M. lupulina* | | *T. arvense* | | *T. dubium* | |  |
|  | *X (Plim-Bal)* | *plX (Plim-Bal)* | *X (Plim-Bal)* | *plX (Plim-Bal)* | *X (Plim-Bal)* | *plX (Plim-Bal)* | *X (Plim-Bal)* | *plX (Plim-Bal)* | *X (Plim-Bal)* | *plX (Plim-Bal)* |  |
| Leaf area | **0.649 **** | 0.013 | **0.607 **** | -0.010 | **1.283 **** | -0.066 | **0.975 **** | 0.081 | **0.989 **** | -0.134 |  |
| SLA |  |  | **-0.352 *** | -0.100 |  |  |  |  |  |  |  |
| LDMC |  |  |  |  |  |  |  |  |  |  |  |
| SPAD | **0.542 **** | 0.043 |  |  | 0.917 | 0.689 | **0.893 *** | -0.207 | **0.711 **** | -0.197 |  |
| RMR |  |  | **-0.323 *** | -0.132 | -0.124 | 0.368 | -0.318 | -0.132 | **-0.562 *** | 0.210 |  |
| Root length | **0.659 **** | -0.057 | **0.523 **** | 0.024 |  |  | **0.943 *** | 0.104 | **0.984 **** | 0.058 |  |
| SRL |  |  |  |  |  |  |  |  | **-0.667 *** | 0.234 |  |
| Surface area | **0.669 **** | **-0.095 *** | **0.535 **** | 0.121 |  |  | **0.990 **** | 0.021 | **1.106 **** | -0.165 |  |
| PME activity | **-0.492 **** | -0.010 | **-0.483 **** | -0.195 |  |  | **-0.843 *** | 0.302 | -0.588 | -0.033 |  |
|  |  | | | | | | | | | | |
|  | Across environment analyses: P limited versus balanced treatment | | | | | | | | | | |
| ***Non-plastic traits*** | Common | | Common | | Common | | Common | | Common | |  |
|  | *B. japonicus* | | *H. murinum* | | *M. lupulina* | | *T. arvense* | | *T. dubium* | |  |
|  | *X (Plim-Bal)* | *plX (Plim-Bal)* | *X (Plim-Bal)* | *plX (Plim-Bal)* | *X (Plim-Bal)* | *plX (Plim-Bal)* | *X (Plim-Bal)* | *plX (Plim-Bal)* | *X (Plim-Bal)* | *plX (Plim-Bal)* |  |
| Leaf area |  |  |  |  |  |  |  |  |  |  |  |
| SLA | -0.126 | -0.034 |  |  | -0.213 | -0.077 | -0.556 | -0.269 | 0.019 | 0.154 |  |
| LDMC | -0.215 | 0.104 | 0.262 | -0.055 | -0.250 | 0.414 | 0.007 | -0.023 | 0.116 | -0.195 |  |
| SPAD |  |  | 0.238 | -0.077 |  |  |  |  |  |  |  |
| RMR | 0.007 | 0.054 |  |  |  |  |  |  |  |  |  |
| Root length |  |  |  |  | 1.522 | -0.541 |  |  |  |  |  |
| SRL | **-0.321 *** | -0.018 | -0.191 | -0.071 | -4.286 | -4.122 | -0.541 | -0.310 |  |  |  |
| Surface area |  |  |  |  | **6.741 *** | 0.395 |  |  |  |  |  |
| PME activity |  |  |  |  | -0.160 | -0.036 |  |  |  |  |  |

**Supplementary Table S8** - *continued*

|  | Across environment analyses: N limited versus balanced treatment | | | | | | | |
| --- | --- | --- | --- | --- | --- | --- | --- | --- |
|  | Invasive | | Invasive | | Invasive | | Invasive | |
|  | *A. sterilis* | | *E. ciliatum* | | *H. jubatum* | | *L. angustifolius* | |
| ***Plastic traits*** | *X (Nlim-Bal)* | *plX (Nlim-Bal)* | *X (Nlim-Bal)* | *plX (Nlim-Bal)* | *X (Nlim-Bal)* | *plX (Nlim-Bal)* | *X (Nlim-Bal)* | *plX (Nlim-Bal)* |
| Leaf area |  |  |  |  | **0.168 **** | 0.007 |  |  |
| SLA |  |  |  |  |  |  |  |  |
| LDMC |  |  |  |  |  |  | 0.070 | -0.025 |
| SPAD | 0.015 | 0.006 | 0.072 | -0.037 | **0.146 **** | 0.008 | 0.097 | -0.070 |
| RMR |  |  | 0.018 | 0.034 |  |  |  |  |
| Root length | 0.050 | 0.016 |  |  |  |  |  |  |
| SRL | -0.027 | -0.046 | -0.058 | -0.009 |  |  |  |  |
| Surface area | 0.044 | 0.033 | **0.143 **** | -0.006 |  |  |  |  |
| PME activity | -0.012 | -0.021 | 0.077 | 0.064 | **0.137 **** | -0.012 | 0.093 | 0.045 |
|  |  |  |  |  |  |  |  |  |
|  | Across environment analyses: N limited versus balanced treatment | | | | | | | |
|  | Invasive | | Invasive | | Invasive | | Invasive | |
|  | *A. sterilis* | | *E. ciliatum* | | *H. jubatum* | | *L. angustifolius* | |
| ***Non-plastic traits*** | *X (Nlim-Bal)* | *plX (Nlim-Bal)* | *X (Nlim-Bal)* | *plX (Nlim-Bal)* | *X (Nlim-Bal)* | *plX (Nlim-Bal)* | *X (Nlim-Bal)* | *plX (Nlim-Bal)* |
| Leaf area | 0.043 | 0.012 | **0.168 **** | 0.030 |  |  | **0.168 **** | -0.077 |
| SLA | -0.044 | 0.020 | -0.074 | 0.053 | -0.029 | 0.040 | -0.037 | -0.022 |
| LDMC | 0.002 | -0.049 | **0.146 **** | 0.064 | 0.015 | 0.048 |  |  |
| SPAD |  |  |  |  |  |  |  |  |
| RMR | 0.004 | 0.016 |  |  | -0.043 | -0.009 | **0.131 *** | -0.010 |
| Root length |  |  | **0.120 *** | 0.006 | 0.097 | -0.012 | 0.051 | 0.009 |
| SRL |  |  |  |  | -0.026 | 0.021 | **-0.130 **** | -0.032 |
| Surface area |  |  |  |  | **0.114 *** | 0.010 | 0.065 | -0.022 |
| PME activity |  |  |  |  |  |  |  |  |
|  |  |  |  |  |  |  |  |  |
|  | Across environment analyses: P limited versus balanced treatment | | | | | | | |
|  | Invasive | | Invasive | | Invasive | | Invasive | |
|  | *A. sterilis* | | *E. ciliatum* | | *H. jubatum* | | *L. angustifolius* | |
| ***Plastic traits*** | *X (Plim-Bal)* | *plX (Plim-Bal)* | *X (Plim-Bal)* | *plX (Plim-Bal)* | *X (Plim-Bal)* | *plX (Plim-Bal)* | *X (Plim-Bal)* | *plX (Plim-Bal)* |
| Leaf area | **0.425 **** | -0.013 | **0.847 **** | 0.057 | **0.704 **** | -0.003 | 0.097 | 0.021 |
| SLA |  |  | -0.408 | -0.034 | **0.466 **** | 0.149 | 0.004 | 0.071 |
| LDMC |  |  | **0.699 **** | 0.376 | **-0.478 **** | 0.034 | -0.031 | 0.046 |
| SPAD | **0.388 **** | -0.008 | **0.789 **** | -0.027 |  |  |  |  |
| RMR | **-0.271 *** | 0.109 | **-0.739 **** | 0.094 | **-0.342 *** | 0.060 |  |  |
| Root length | **0.418 **** | -0.075 | **0.826 **** | 0.038 | **0.673 **** | 0.095 | **0.140 **** | -0.047 |
| SRL |  |  |  |  |  |  |  |  |
| Surface area | **0.407 **** | -0.041 | **0.743 **** | -0.067 | **0.672 **** | -0.077 |  |  |
| PME activity | **-0.312 **** | 0.038 | -0.474 | 0.122 | **-0.608 **** | -0.098 |  |  |
|  |  | | | | | | | |
|  | Across environment analyses: P limited versus balanced treatment | | | | | | | |
|  | Invasive | | Invasive | | Invasive | | Invasive | |
|  | *A. sterilis* | | *E. ciliatum* | | *H. jubatum* | | *L. angustifolius* | |
| ***Non-plastic traits*** | *X (Plim-Bal)* | *plX (Plim-Bal)* | *X (Plim-Bal)* | *plX (Plim-Bal)* | *X (Plim-Bal)* | *plX (Plim-Bal)* | *X (Plim-Bal)* | *plX (Plim-Bal)* |
| Leaf area |  |  |  |  |  |  |  |  |
| SLA | -0.131 | -0.004 |  |  |  |  |  |  |
| LDMC | -0.111 | 0.071 |  |  |  |  |  |  |
| SPAD |  |  |  |  | **0.425 *** | -0.086 | 0.029 | -0.031 |
| RMR |  |  |  |  |  |  | 0.079 | -0.050 |
| Root length |  |  |  |  |  |  |  |  |
| SRL | -0.162 | -0.024 | 0.326 | 0.138 | -0.003 | 0.042 | -0.056 | 0.033 |
| Surface area |  |  |  |  |  |  | **0.149 **** | 0.020 |
| PME activity |  |  |  |  |  |  | -0.063 | -0.015 |
